# Digital Contact Tracing Apps for COVID-19: Development of a Citizen-Centered Evaluation Framework

**DOI:** 10.2196/30691

**Published:** 2022-03-11

**Authors:** Damyanka Tsvyatkova, Jim Buckley, Sarah Beecham, Muslim Chochlov, Ian R O’Keeffe, Abdul Razzaq, Kaavya Rekanar, Ita Richardson, Thomas Welsh, Cristiano Storni, COVIGILANT Group

**Affiliations:** 1 Lero, Science Foundation Ireland Research Centre for Software University of Limerick Limerick Ireland; 2 Department of Computer Science and Information Systems University of Limerick Limerick Ireland; 3 Interaction Design Centre University of Limerick Limerick Ireland; 4 see Acknowledgments for contributors

**Keywords:** COVID-19, mHealth, digital contact tracing apps, framework, evaluation, mobile health, health apps, digital health, contact tracing

## Abstract

**Background:**

The silent transmission of COVID-19 has led to an exponential growth of fatal infections. With over 4 million deaths worldwide, the need to control and stem transmission has never been more critical. New COVID-19 vaccines offer hope. However, administration timelines, long-term protection, and effectiveness against potential variants are still unknown. In this context, contact tracing and digital contact tracing apps (CTAs) continue to offer a mechanism to help contain transmission, keep people safe, and help kickstart economies. However, CTAs must address a wide range of often conflicting concerns, which make their development/evolution complex. For example, the app must preserve citizens’ privacy while gleaning their close contacts and as much epidemiological information as possible.

**Objective:**

In this study, we derived a compare-and-contrast evaluative framework for CTAs that integrates and expands upon existing works in this domain, with a particular focus on citizen adoption; we call this framework the Citizen-Focused Compare-and-Contrast Evaluation Framework (C^3^EF) for CTAs.

**Methods:**

The framework was derived using an iterative approach. First, we reviewed the literature on CTAs and mobile health app evaluations, from which we derived a preliminary set of attributes and organizing pillars. These attributes and the probing questions that we formulated were iteratively validated, augmented, and refined by applying the provisional framework against a selection of CTAs. Each framework pillar was then subjected to internal cross-team scrutiny, where domain experts cross-checked sufficiency, relevancy, specificity, and nonredundancy of the attributes, and their organization in pillars. The consolidated framework was further validated on the selected CTAs to create a finalized version of C^3^EF for CTAs, which we offer in this paper.

**Results:**

The final framework presents seven pillars exploring issues related to CTA design, adoption, and use: (General) Characteristics, Usability, Data Protection, Effectiveness, Transparency, Technical Performance, and Citizen Autonomy. The pillars encompass attributes, subattributes, and a set of illustrative questions (with associated example answers) to support app design, evaluation, and evolution. An online version of the framework has been made available to developers, health authorities, and others interested in assessing CTAs.

**Conclusions:**

Our CTA framework provides a holistic compare-and-contrast tool that supports the work of decision-makers in the development and evolution of CTAs for citizens. This framework supports reflection on design decisions to better understand and optimize the design compromises in play when evolving current CTAs for increased public adoption. We intend this framework to serve as a foundation for other researchers to build on and extend as the technology matures and new CTAs become available.

## Introduction

The global coronavirus pandemic (COVID-19) calls for rapid measures to monitor and control the spread of the virus. Contact tracing is one of the measures adopted by health authorities. This approach has already been used with a certain level of success for other dangerous illnesses such as tuberculosis [1] and Ebola [2]. As part of the contact tracing effort in the COVID-19 pandemic, the deployment of mobile apps, and their potential in collecting, storing, and sharing citizens’ contact tracing data have been examined, with early studies showing favorable results [3,4]. These studies have contributed to the impetus for using digital contact tracing apps (CTAs), and many CTAs have been developed for nations’ use to facilitate community-based disease surveillance [5].

The application of CTAs in real-world settings has provoked numerous discussions regarding their design [6-9], concerns about the security and privacy of CTA data, and the barriers for their widespread acceptance and adoption by citizens [10,11]. Reflecting these discussions, CTA evaluation frameworks have emerged that specifically focus on different aspects such as the assessment of contact tracing architectures [12], sociotechnical issues [13], privacy [14,15], ethical and legal challenges [16], feasibility and effectiveness [17], usability [18], and essential attributes [19,20].

In the context of such fragmentation, a legitimate concern is for a more comprehensive evaluation framework that would encompass a variety of different aspects of CTAs, pertinent to the adopting citizens, and which would enable decision-makers (eg, developers, health authorities) to assess and possibly improve their designs. This concern drives our research question: *how to devise and organize a framework to enable a more comprehensive assessment of current CTAs, supporting the work of decision-makers (eg, developers, health authorities) in the development and evolution of CTAs, potentially increasing adoption?* In this paper, we address this question by proposing a Citizen-Focused Compare-and-Contrast Evaluation Framework for CTAs (C^3^EF), which we derived by holistically bringing together existing works on the evaluation of CTAs and mobile health (mHealth) apps, and iteratively grounding and stress-testing our derivations with a number of current CTAs. 

The framework proposed here is focused more on the apps themselves than on the apps’ embedding in national health systems (as another important perspective [21]). The framework is organized to help in the assessment and improvement of existing CTA solutions through a taxonomy of 7 pillars that focus on clustered attributes: (General) Characteristics, Usability, Data Protection, Effectiveness, Transparency, Technical Performance, and Citizen Autonomy. This article introduces the C^3^EF and presents its derivation over several iterations. As we present the framework, we will illustrate the framework’s application to a selection of existing CTAs, showing how the framework can be used to assess and possibly improve aspects of CTA design.

The next section presents an overview of how we developed our framework. This is followed by an overview and discussion of the C^3^EF framework itself. Our Discussion presents a summary of our contributions, limitations of this study, and future work.

## Methods

### Review and Framework Derivation

To accommodate the high-complexity and multidisciplinary nature of CTAs evaluation for wide societal adoption, we used an iterative approach combining a literature review and expert opinions with an empirical application of the derived attributes, and their associated questions, to the evaluation of actual CTAs. Within this approach, the multidisciplinary nature of the framework was specifically handled by a progressive “segmentation,” where 10 domain experts within the research team were allocated responsibility for individual parts of the framework.

**Figure 1:**
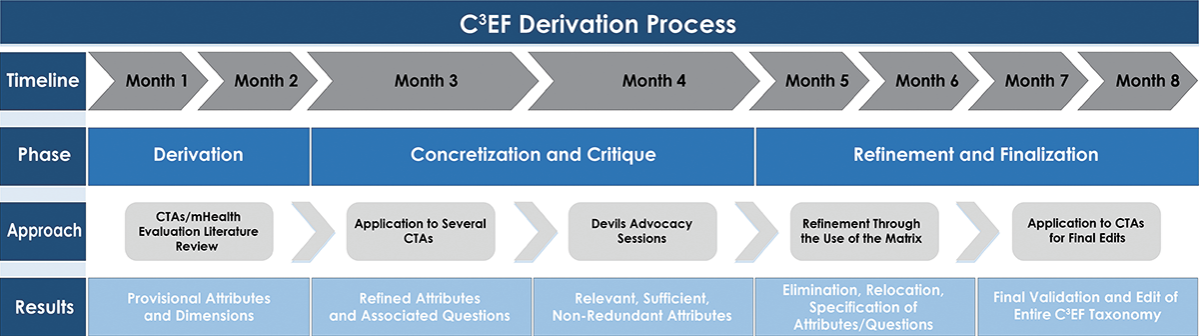
Phases and deliverables in the development of our Citizen-Focused Compare-and-Contrast Evaluation Framework (C^3^EF) for contact tracing apps (CTAs). mHealth: mobile health.

This methodology is portrayed in Figure 1, which shows the three main phases over the period of 8 months, starting in June 2020. Phase 1 focused on initial derivation (for months 1 and 2), phase 2 focused on concretization and critique of the prototype framework derived in phase 1 (months 3 and 4), and phase 3 focused on final refinements (months 5 to 8).

### Phase 1: Derivation

#### Literature Review

This phase was based on a “critical (literature) review” [22] of relevant areas. Here, iterative refinement/evaluation is used to focus in on more optimal search parameters, search databases, the search string, and inclusion/exclusion criteria based on the initial research question. Ultimately, to obtain a holistic perspective, this resulted in us focusing on:

1. CTAs: these included peer-reviewed literature on existing evaluation frameworks, grey publications discussing design characteristics and functionality, and design guidelines/EU regulations for development of effective/appropriate digital CTAs.
2. Mobile apps: this included the most recent evaluation frameworks for mobile apps, evaluation frameworks for mHealth apps, accessibility principles for mobile apps, universal design (UD) for the apps, and taxonomies of usability.

To reflect this broad focus, the search sources employed were:

1. Electronic databases to search academic texts: Google Scholar, Elsevier, ACM Digital Library, Sage, IEEE Xplore, and Springer.
2. Searches of web-based grey literature (using Google).
3. Consulting the reference lists of the selected articles to identify further relevant studies, following the systematic “backward snowballing” protocol proposed by Wohlin et al [23]. This allowed us to use the original sources to recursively increase our existing set of articles. “Forward snowballing” [23] was not used, based on the relatively recent appearance of CTA-specific literature.

The search string derived from the critical review was “evaluation frameworks” AND “digital contact tracing applications” AND “COVID-19” OR “mobile applications” OR “mHealth applications” OR “accessibility” OR “universal design” OR “usability” OR “taxonomies” OR “Data protection” OR “GDPR” OR “security threats.” Articles written in English and published between 2010 and 2020 were reviewed. Articles offering evaluation frameworks were selected as well as articles discussing particular aspects, qualities, or characteristics of CTAs/mHealth apps. Inclusion was assessed by reading the abstract, and, in cases where the abstract was insufficient, by reading their introduction and conclusion. All 10 researchers from the team were involved in the search and selection of the sources, with marginal papers being discussed for relevance in dedicated group meetings.

With this search strategy, we identified 44 relevant sources (a full list is available in Multimedia Appendix 1 [6,7,9,13-17,24-59]). Twenty-one of these were distinct frameworks focusing on particular aspects of CTAs, 13 provided regulations and guidelines for the design or evaluation of CTAs or mHealth apps, and 10 others described important characteristics for CTAs. From these sources, one of the researchers extracted an initial set of 111 attributes, representing a pool of attributes to be used for the derivation of the first iteration of the framework. Again, these were reviewed in a group meeting, where more marginal attributes were debated, but all ultimately persisted. 

We performed a cluster analysis of this initial attribute list, which was aimed at identifying overlaps and affinities, and at grouping them into thematic areas. That is, we focused on constructing an “information architecture” of categories, but not a “navigation structure” between categories, as described by Righi et al [60]. We then juxtaposed our identified areas with those explicitly provided in the papers directed at frameworks and taxonomies for CTAs [13,14,17,24-31,58,59], and we ended up identifying 6 evaluation areas, which we call pillars: *Usability*, *Data Protection*, *Effectiveness*, *Transparency*, *Technical Performance,* and the degree of *Autonomy* the app provides to downloading citizens. To uniquely identify an app and report on its nonevaluative characteristics, “*General Characteristics*” was also added. Multimedia Appendix 1 offers a full list of the included papers, a table of the extracted attributes and categories from the selected papers, and how we grouped them into our resulting 6 pillars. At this stage, the project team was divided into domain expert subgroups, one for each pillar, working on their specific development and further refinement. Overall, these subgroups reflected a range of competencies such as software engineering, human-computer interaction, security, and data protection.

#### Usability

Usability refers to the ability of the CTAs to be easy to use and understood. We prioritized concerns of usability as the project centered around increasing adoption by citizens, and therefore understanding its usability for target audiences was essential. We derived the initial attributes after a review of CTA evaluation frameworks [14-17,58], and from the usability frameworks for mobile apps/mHealth apps, accessibility, and UD literature [27,29,31,44-50]. Other sources that informed our deliberations were those discussing usability standards [61-63] in general and EU design requirements [55]. Accessibility was included as a high-level attribute under usability, and was mostly derived from the EU directive Accessibility EN 301 549 [30], from where we took initial requirements and checked them against the Web Content Accessibility Guidelines [56] to formulate probing questions attached to our identified attributes. Frameworks for designing touchscreen interfaces for children [52], evaluating apps for children [53], and General Data Protection Regulations (GDPR) regulation for minors [54] were also consulted. An early report of the work in this pillar is available [64].

#### Data Protection

Data Protection was chosen to accommodate societal concerns of privacy and security inspired by similar attributes in related works [28]. Although it has been noted for its complexity [65], we selected GDPR as our reference for the development of the Data Protection pillar: the GDPR of the European Union [66] is currently considered the foremost data protection legislation worldwide for protecting the rights of the individual. We retrieved the initial attributes and preliminary questions from national legal interpretations [67,68] for data-focused concerns. Since this approach excludes wider organizational attributes (now found, for example, within our Transparency pillar) and system-oriented goals such as information security, we developed a novel risk-based approach to compare the system security of CTAs, based on a review of the related literature in mobile app GDPR evaluation [65,69-76].

#### Effectiveness

Effectiveness measures how successful an app is in terms of the accuracy of its contact tracing, the COVID-restraining impact of the app over a jurisdiction, and the app’s popularity with citizens. Concerns include detecting and sharing close contacts, providing relevant information to citizens, and assessing their reactions to that information. This pillar was informed by drawing and expanding on the definition of effectiveness in CTAs, provided by Lueks et al [77], and by considering Vokinger et al’s [58] framework, which also explicitly tackles this concern.

#### Transparency

While transparency is officially a subset of GDPR, a separate Transparency pillar was created in the framework to consider wider aspects of transparency not specifically related to functionality. For example, while the GDPR approach to transparency considers specific data stores (such as locally stored contact information), transparency concerns such as the availability of a privacy policy or the open-sourcing of the source code would not fit into that approach. In other words, “transparency” in this context concerns how open the developing organization is with respect to its internal processes and artifacts. The initial attributes and their questions were formulated by extending our interpretation of GDPR [66], and considering already existing taxonomies [25,28].

#### Technical Performance

The Technical Performance pillar captures the efficiency of the contact tracing. Particularly, the Technical Performance pillar focuses on system resource utilization and execution speed, as these aspects impact use. The relevance of this pillar can be seen in how, for example, battery issues with the Exposure Notification Service provided by Google [78] and incorporated into the national Contact Tracker app in Ireland, caused battery issues over only one weekend and caused a large fall-off in app retention by the public [79]. The attributes for the Technical Performance pillar can be divided into resource utilization–related performance (eg, CPU/disk/memory usage) and efficiency-related performance (eg, response time). Because COVID-19 tracing apps are usually complex software systems (with dedicated front-end and back-end subsystems), the attributes can be applied to both subsystems respectively. The initial attributes for this pillar were derived from the “Performance Efficiency” category of ISO/IEC 25010, a software engineering quality model. The model is a standard for assessing characteristics of software systems and is widely applicable in software engineering.

#### Citizen Autonomy

Citizen Autonomy focuses on the citizen’s ability to consent and the voluntary nature of the app. Its inclusion was inspired by the work of Gasser et al [16] studying a digital tool’s ethical challenges. It was also based on the “User control/self-determination” domain in Vokinger et al’s [58] assessment framework for (COVID-19) CTAs and the “autonomy” category in the checklist proposed by van Haasteren et al [28]. In these works, the authors focused on users’ (existing) “data protection” concerns, which are mostly handled by our Data Protection pillar, but we wanted to extend the scope to specifically cover *initial* data access. Hence, this pillar focuses on a series of specific attributes that assess citizens’ control over the app’s access to phone functionalities such as the camera, microphone, and GPS.

#### (General) Characteristics

“General Characteristics” refers to characteristics that are nonevaluative, but serve to distinguish the app from others and other versions of the app. Thus, the static information captured by the Characteristics pillar acts as a necessary first step to conducting the more in-depth compare-and-contrast evaluation found in the other pillars. An initial set of distinguishing characteristics was derived by examining three CTAs: SwissCovid (Switzerland) [80], Apturi Covid (Latvia) [81], and Immuni (Italy) [82], and related data retrieved from their AppStore, Google Play, and app home websites. Next, we analyzed the Google and Apple Exposure Notification (GAEN) application programming interface (API)/framework [78] made available for use on Apple and Android devices, and the Decentralized Privacy Preserving Proximity Tracing (DP-3T) protocol [83,84] that inspired Google’s API. We expanded our list of attributes further through a review of contact tracing protocols and frameworks listed on the Wikipedia COVID-19 Apps page [85]. Finally, we incorporated the literature review of app/mHealth app evaluations (see the grey literature in Multimedia Appendix 1 [32-39]).

At the end of the work described above, our initial list of 111 attributes had grown to 139 organized under 7 pillars.

### Phase 2: Concretization and Critique

#### Test of the Framework Against Five CTAs

In the second phase of development, our provisional framework was tested against five CTAs that could be downloaded and activated in Ireland: Health Service Executive (HSE) COVID Tracker app (Ireland) [86], PathCheck SafePlaces (Massachusetts Institute of Technology [MIT], United States) [87], NOVID (United States) [88], Corona-Warn (Germany) [89], and Aman (Jordan) [90].

The two core considerations were to assess if the attributes could produce useful evaluation information on the CTA and how that information could be feasibly obtained. In this phase, feasibility concerns were sometimes overridden by the perceived importance of the information provided when probing a specific attribute. For example, the “number of people alerted-early to their close contact status, who then go for testing,” seems like one of the core “Effectiveness” measures for CTAs. However, identifying this number involves a much wider information-gathering and reporting effort than is normally available from the app itself, and so could be quite difficult to assess [21] (ie, members of the public would need to inform authorities when they turn up for testing that they have done so based on an alert issued to them by the app, and that would need to be recorded on a national health system that ideally integrates back with the CTA).

The selected five apps reflected a *broad range* of approaches, as illustrated by their different *lead bodies*, and the different *data protection philosophies* underpinning them. In terms of “broad range,” the Republic of Ireland’s app has provided the basis for apps in other jurisdictions, both in Europe and the United States [79]. In terms of “lead bodies,” these apps come predominantly from national health services, but PathCheck SafePlaces is an MIT-led initiative [91] and NOVID is crowd-sourced, originating from Carnegie Mellon University [88]. In terms of *data protection philosophies*, two of these apps originate in GDPR jurisdictions, but two originate from the United States, and one originates from Jordan.

The process followed in this phase was that the domain experts would apply their pillar to the five chosen apps to stress-test the ability of the framework to identify criticalities and key differences among apps. For each of the identified attributes, they formulated appropriate questions, and assessed the answers obtained to see if the attributes and the related questions had evaluative merit. Attributes were added where necessary, sometimes merged or reorganized. For example, in *General Characteristics*, the version number was identified in this phase as an important identifier, as CTAs, like other apps, tend to receive regular updates. Likewise, in *Effectiveness*, the effort/speed with which close contacts are alerted by CTAs was also identified as important. In contrast, *Usability* made sure that “accessibility” aspects were treated specifically and separately from more general usability and interaction aspects, which resulted in reorganizing some of the subattributes.

This concretization was sometimes complemented and reinforced with further, targeted reviews of the literature where deemed necessary.

#### Devil’s Advocates Sessions

As the pillars were developed independently, a series of meetings across the expert subgroups were held to retain a wider perspective. Specifically, the meetings were organized so that domain experts, who were testing and refining a specific pillar, presented and defended their work to the other team members (see Table 1) who dissected the pillar and questioned its attributes under the headings of*:*

- *Relevance and Sufficiency*, where the team was encouraged to ask questions such as “why is this important?” and “what else might be important?”;
- *Specificity*, where domain experts were encouraged to hypothetically answer each of the associated questions in the pillar and to (thus) probe it for any ambiguity; and
- *Cross-checking* with their own pillars to identify possible overlaps in the framework.

**Table 1:** Distribution of team members as pillar owners and devil’s advocates in phase 2.

Pillar name	Pillar owner(s)	Devil’s advocate(s)
(General) Characteristics	IO and SB	JB
Usability and Accessibility	CS, IR, and DT	IO and JB
Data Protection	TW	KR
Effectiveness	AR	DT
Technical Performance	MC	KR
Transparency	KR	MC
Citizen Autonomy	JB	IR

These meetings were in the form of “devil’s advocate” sessions (7 in total), where 1 participant actively tried to identify/exaggerate flaws in the current attributes. This is because such an approach has been shown to increase the “accuracy of group solutions” [92]. The activity highlighted a number of changes mostly concerned with clarifying potential overlaps or redundancies, clarifying terminology and questions, and improving organization. At the end of the grounding and critiquing exercises, we ended up with a total of 163 “grounded” attributes and an initial formulation of 199 related questions. Additionally, we identified some cases to be discussed by the entire team during the third and final phase of our development.

### Phase 3: Refinement and Finalization

To support the refinement work of the entire team (especially in consideration of the remote work environment demanded by COVID-19), we created a cross-pillar analysis matrix (Multimedia Appendix 2), which listed the ordered attributes and subattributes for each of the 7 pillars, assigning a unique identifier to each attribute (eg, the first General Characteristic attribute was assigned the identifier C01) and a color code to each pillar. To further support understanding of the attributes and to clarify their evaluative merit, we decided to also add sample answers (based on our grounding analysis of the five apps). We discussed the difficult cases identified in the previous phase and noted further (relevancy, sufficiency, specificity, and redundancy) issues for each attribute over a total of 15 refinement sessions around this cross-pillar matrix. The identified changes were progressively included in the framework, and 9 of the 10 authors were involved. For instance, the framework probes *Citizen Autonomy* in terms of whether there is an official discussion forum for citizens using the app and whether that forum can be used to prompt change (CA01, CA02; see Multimedia Appendix 2). It was noted that these overlap with attribute C16, a *Characteristic* attribute that probed the form of technical support, and U73, a *Usability* attribute probing the existence of interactive assistance for technical support or any other mechanism to submit feedback on technical issues, bugs, and errors detected. A reorganization was proposed, deleting CA11 (redundant with CA01 and CA02); changing C16 from “*Does the app offer technical support?*” into “*What form of technical support is available for the users, to include synchronous and asynchronous support?*”; and simplifying CA02 by removing its reference to *any other mechanism (to obtain technical support)*, as this was covered by the new phrasing of C16.

At the end of our 15 sessions, the refined pillars were applied to the newest versions of two of the five apps employed in the “grounding” phase (HSE’s COVID Tracker [86] and NOVID [88]) to systematically double-check all attributes, questions, and answers so that we could either confirm or implement final edits. The main goal was to make sure the questions were clear and understandable. A number of other apps were also assessed less systematically to the same end.

At the end of this last test, our consolidated framework was restructured to 161 attributes and 180 related questions (with sample answers), which now had internal consistency and no overlap. Graphical visualizations of the refined pillars and their structures were also generated (Multimedia Appendix 3).

The consolidated framework (with 7 pillars and 161 attributes) was presented for feedback to medical researchers and practitioners from the wider “COVIGILANT” group, the (Irish) Department of Health, and the (Irish) HSE’s “App Advisory Group,” which included representative from Nearform, the company charged with creating the Irish national CTA. Likewise, informal discussions were held around the Effectiveness pillar with the European Centre for Disease Control (CDC), all serving to suggest a number of minor edits and tweaks to create the final version of the C^3^EF for CTAs, as presented here.

At this stage, we also created a web-based application [93] to make our framework available in the form of an online survey. This acts as a demonstrator of our framework, but it has been devised to possibly assist relevant stakeholders of CTAs in independently evaluating their work and/or to share any feedback with us. This online tool offers visual overviews of the framework and gives access to the entire framework. With the depth and range of questions included in our C^3^EF, the evaluation process may appear daunting and time-consuming. Consequently, we decided to provide access to individual pillars to enable breaking down the assessment, and allow stakeholders to select and prioritize their own assessment focus.

## Results

### Overview of the C^3^EF Framework

In this description of our final framework, we define each pillar and provide an overview of its specific attributes, subattributes, and questions. We then offer a selection of sample questions and answers to illustrate how we used the framework to evaluate, compare, and contrast CTAs, and how this could be conductive of possible improvements in the apps, as questions often probe the desirable or best practice options. Table 2 offers a top-level view of the 7 pillars and the high-level attributes. (General) Characteristics is presented first, as it provides important contextualization/identification information for the other six pillars.

**Table 2:** The 7 pillars with their first- and second-level attributes (only).

First-level attributes		Second-level attributes
**Characteristics Pillar**		
	1. General characteristics	
		Name of app
		Country
		Current versions
		Language support
		Age of users
	2. Availability	
		Internet connectivity: app (other)
		Platform dependency
	3. Organizational reputation	
		App status
		Development
	4. App content	
		Processing overview
		Sensor employed
		App running state
		Contact tracing definition
		App data
		App permissions
		Notification method
		Diagnosis status
**Usability Pillar**		
	1. Subjective satisfaction	
		Rating
		Motivations for high/low scores
	2. Universality	
		Accessibility
		Cultural universality
	3. Design effectiveness	
		Completeness
		Configurability
		User interface
		Helpfulness
	4. User interaction	
		Efficiency
		Robustness
		Clarity of interaction with elements
		Consistency of interaction with elements
		Alerts and notifications messages
	5. Ongoing app evaluation	Frequency of upgrade

**Data Protection Pillar**		
	1. Security	
		STRIDE^a^ taxonomy/vulnerabilities
		CT^b^-specific threats
		Software architecture security
		SDLC^c^ and security
	2. GDPR^d^	
		Preliminaries
		GDPR principles
		Rights
**Effectiveness Pillar**		
	1. Effective reporting	
		Detecting close contacts
		Reporting positive close contacts
		Reporting all close contacts
		Reporting hotspots
	2. Effective results	
		Users who share their data
		Number of (additional) contacts/week found
		Number of those contacts found positive
		Relative effort per contact found versus manual CT
	3. Effective engagement	
		Population uptake
		Population retention
		Population engagement
**Transparency Pillar**		
	1. App transparency	
		App purpose
		App permission
	2. User participation	App participation knowledge
	3. Data transparency	
		Minimization, gathering, storing, accessibility, etc
		GDPR applicability
		Life cycle
**Technical Performance Pillar**		
	1. Speed	Response time (frontend)
	2. Efficiency	Response time
	3. Consumption	
		Battery
		Disk space
	4. Resource/troubleshooting and trust	
		CPU/memory usage
		Bandwidth usage
		Throughput (backend)
**Citizen Autonomy Pillar**		
	1. App discussion authority	
		Official discussion forums
		Empowered moderators
	2. Phone functionality	
		GPS access
		Bluetooth
		ENS^e^ access
		Notifications
		Microphone
	3. Data control	
		Data upload authority
		Uploaded data location visibility

^a^STRIDE: Spoofing, Tampering, Repudiation, Information Disclosure, Denial of Service, and Elevation of Privilege.

^b^CT: contact tracing.

^c^SDLC: Software Development Life Cycle.

^d^GDPR: General Data Protection Regulation.

^e^ENS: Enhanced Network Selection.

### Characteristics

Characteristics captures nonjudgmental criteria and factual information that are important to identify and differentiate a given app and its main functionalities. The App Characteristics pillar is organized according to four headings: *General Characteristics, Availability, Organizational Reputation,* and *App Content* (Table 2). Under these headings, there are a total of 25 specific questions that elaborate upon these app characteristics, all of which can be answered by direct inspection of the working app, through the information available on Google Play and Apple Store, or through the developer website.

*General Characteristics* captures four high-level app attributes, including the name of the app and the country. *Availability* looks at connectivity and platform dependency. The first aspect of availability questions whether an internet connection is needed to use the app, as some apps appear to require an internet connection even if they do not use the internet or location-based information for their contact tracing (eg, the Jordanian app AMAN [90]). The second questions are related to what platforms (Android/iOS) are supported and the download size.

*Organizational Reputation* looks at the status of the app, including whether the app is national and what official documentation is available. It also considers the organization that developed the app and whether any third-party or partners are involved in its development. This attribute examines the history of development, through an examination of the developers’ prior experience of developing data-sensitive apps, along with evidence of updates, enhancements, and maintenance of the actual product. Even if we are aware that questions concerning organizational reputation (such as history) may disadvantage apps from new startups, we believe that this attribute captures an aspect that contributes to the confidence users will have in app adoption. Finally, the ability for users to ask questions and seek technical support is also probed.

*App Content* refers to what the app includes in terms of functionality and management of information. Definitions of contact tracing are queried, and information is given as to when and how contact tracing notifications are managed, since this may vary from country to country, or even from app to app. This is where key distinctions can be made between apps that use a different approach to contact tracing and notification. For instance, the HSE’s app [86] states that the close-contact notifications will be activated when there is “direct exposure” to a positive case, where “direct exposure” refers to “within two meters for 15 minutes or more.” In contrast, NOVID [88] notifies users when other NOVID users close in their social network are positive cases. The former, a more common approach, supports health authorities in warning citizens that they have been in contact with a positive case, while the latter notifies when the infection is close and aims at warning the citizens ahead of being in contact with the virus. NOVID classifies physical proximity as “near” or “far,” with 6 feet (2 meters) or under being “near” and 12 feet (4 meters) or over being “far.” Definitions of physical proximity in both systems (the HSE’s app and NOVID) are based on parameters for proximity classification that can change in updated versions of the apps, depending on variants and infection events. However, at their core, these apps offer a different benefit to users. NOVID (which is not a national app and thus might be difficult to reach the required critical mass of citizens adopting the system to help it perform at its best) seems to have an obvious advantage from the perspective of citizens’ adoption. However, it would raise some issues under our Data Protection pillar because, even though this approach captures no personalized citizens’ data and so users can be entirely anonymous, the social network data would need to be centrally collected and aggregated, providing indirect, yet not impossible, opportunities to deanonymize the information. We note that while such widely differing approaches make the application of any framework difficult, this attribute highlights the divergence at an elevated level, and many of the attributes proved resilient to this (NOVID’s) different paradigm [94], though not all.

### Usability

Five high-level attributes have been identified under *Usability: Subjective Satisfaction, Universality, Design Effectiveness, User Interaction,* and *Ongoing App Evaluation* (Table 2). Together, they offer the opportunity to ask 86 specific questions about usability aspects of CTAs.

*Subjective Satisfaction* looks at the perceived level of comfort experienced in using the app. As user retention is important in CTAs, this attribute captures how citizens rate their experience in using the app. It includes a rating attribute (1 to 5), and two attributes looking at motivations for high or low scores. To inform our answers to these questions, we typically look at the rating and reviews available on Google Play and Apple Store, although satisfaction could be better captured with longitudinal surveys.

**Figure 2:**
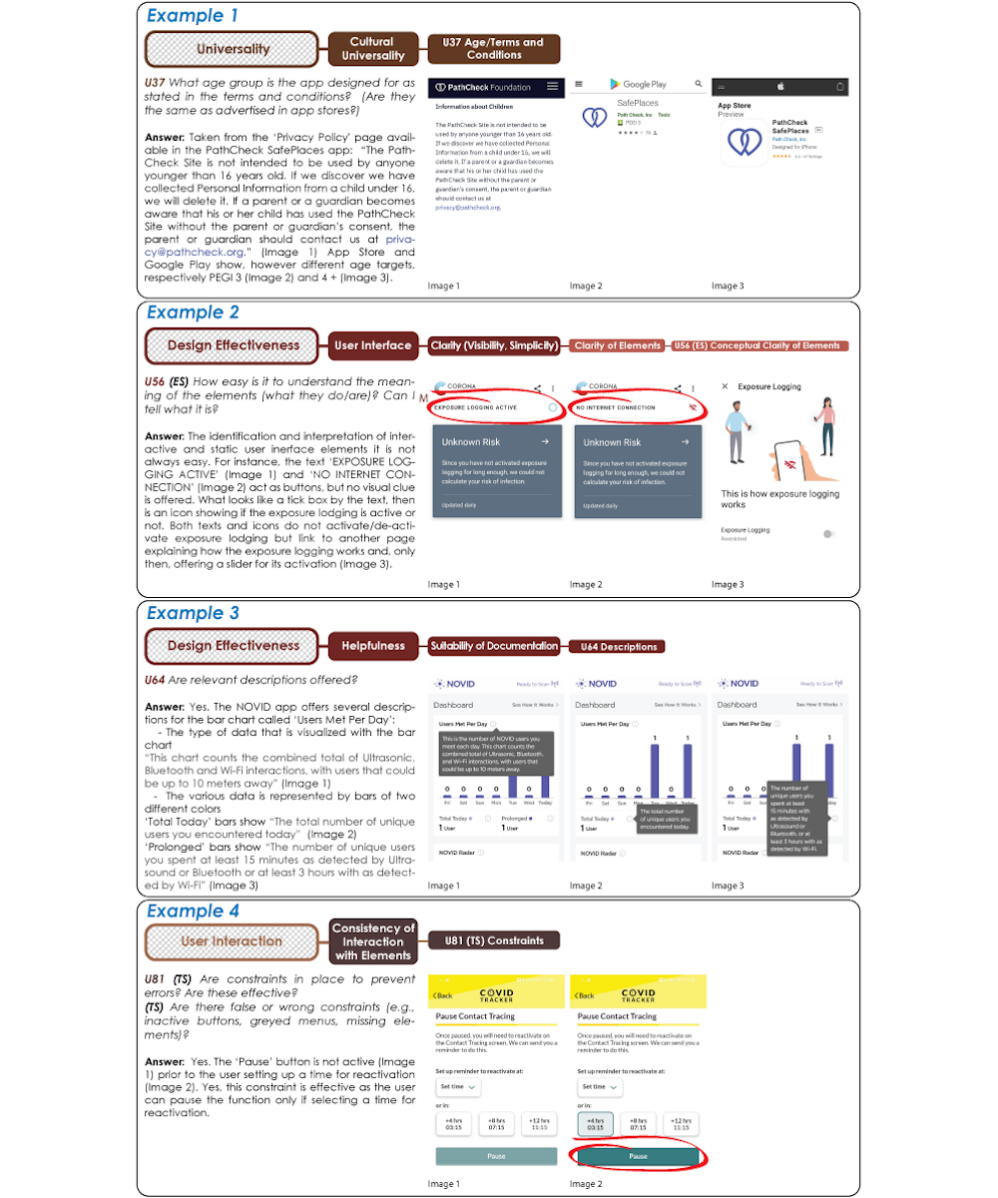
Example 1, PathCheck SafePlaces (United States) [87]: age in "Terms of Use." Example 2, Corona-Warn (Germany) [89]: understandability of interface elements. Example 3, NOVID app (United States) [88]: descriptions offered. Example 4, COVID Tracker app (Ireland) [86]: constraints for preventing errors.

*Universality* addresses population penetration, which is also key in the successful implementation of a CTA strategy. Specifically, universality aims to capture the ability of the app to be used by a variety of different users: users with potential impairments (physical or mental), but also users with different cultures/levels of education or of different ages. The first is captured by the subattribute Accessibility, and the latter is captured by Cultural Universality. Accessibility refers to the quality of being “easy to reach and use,” and it mostly refers to users who might have a form of disability, impairment, or limitation (either mental or physical). We covered three aspects related to Accessibility: *Functional Performance*, *User Interface Elements*, and *Accessible Interactions*. The first two look at the interface and how its elements adhere to general accessibility guidelines and EU regulations [6,29,30,45,46,56]. Questions can be used as a checklist to make sure the app meets basic accessibility requirements. *Accessible Interaction*, the last subattribute under Accessibility, covers aspects such as onboarding (ie, features helping new users understand what the app does and learning how to use it) and the design of interactive elements to support low physical effort (eg, completing a task without scrolling, one-hand use, radio buttons). *Cultural Universality* helps to assess the extent to which the system can be used by different users regardless of their cultural background and beliefs. We developed attributes and questions to cover aspects such as (1) availability of different languages; (2) meanings that are evoked by the name of the app; (3) information on the age groups that the app targets, usually described in the “Terms and Conditions” (see Figure 2, Example 1); and (4) design elements such as logos, colors, national flags, and symbols for expressing cultural conventions.

*Design Effectiveness* covers several aspects concerning the capacity of the system, user interface, and interaction design to provide citizens with the necessary functionalities, options, commands, and supports. This attribute includes four dimensions that are found to be key in conveying the correct utilization of the system and its adaptation to different contexts of use and user preferences: *Completeness*, *Configurability*, *User Interface*, and *Helpfulness*. Completeness was formulated to identify both essential and optional functionalities offered by the app and those features that are not included in the app, but that users (eg, as voiced on online reviews) would like to have. Identification of core and optional functionalities help to identify user tasks that can be carried out by the user in interacting with the CTA interface, and this forms the basis for task-specific questions in our framework, which will come later. The identification of optional and emerging new functionalities is important as CTAs continue to evolve and offer new uses beyond contact tracing (eg, check-in, digital vaccination certificates, travel passes), and our question about optional functionalities allows our framework to incorporate them in the assessment. However, there is a tradeoff is in play here, which our framework can help to capture: offering a number of different functionalities could potentially increase the attractiveness of a CTA, thus resulting in higher adoption and satisfaction, while accommodating more functionalities within the same app might compromise usability (in our case resulting in potential issues identified under our accessibility, design effectiveness, and user interaction attributes). The next attribute (*Configurability*) looks at a variety of aspects concerning the capacity of the system to be personalized in terms of the technology in use (eg, allowing independent activation or deactivation of GPS, Bluetooth, and other technologies) or its design (eg, allowing personalization of visual, acoustic, and haptic feedback). *User Interface* deals with the assessment of the design elements used in the user interface with its *Aesthetic and Attractiveness* (concerned with the look and feel, color palette, and name of the app), *Responsiveness* (concerned with the ability to adapt to different phone models, screen sizes, and operating systems), and *Clarity* and *Consistency* of the design elements. These two last attributes offer *element-specific* subattributes, in that they refer to and evaluate specific elements of the interface and not the app as a whole. An element can vary from a button to a menu, a slider, or a table (see [95] for a full list and glossary of user interface elements).

Our questions explore the apps’ perceptual and conceptual clarity, looking at their visibility, understandability (Figure 2, Example 2), and consistency (again both in terms of how they look and that their meaning is consistent throughout the app). In this sense, we intend for the framework to help to find potential flaws, hindering satisfaction, use, and adoption. However, both *Clarity* and *Consistency* also apply to structures of elements (eg, a button-bar containing buttons) in terms of how elements are logically grouped and whether these logic-based groupings are consistent. The last subattribute of *Design Effectiveness* is *Helpfulness*, looking at the suitability of documentation available to use and understand the app, and whether interactive assistance is available. Subattributes look at the availability of supportive information such as definitions of the terms used (eg, what counts as a contact), descriptions (see Figure 2, Example 3), examples (eg, tutorial, walk-through or explanatory videos showing how to use the app), and the availability of interactive assistance for troubleshooting (eg, chatbots).

**Figure 3:**
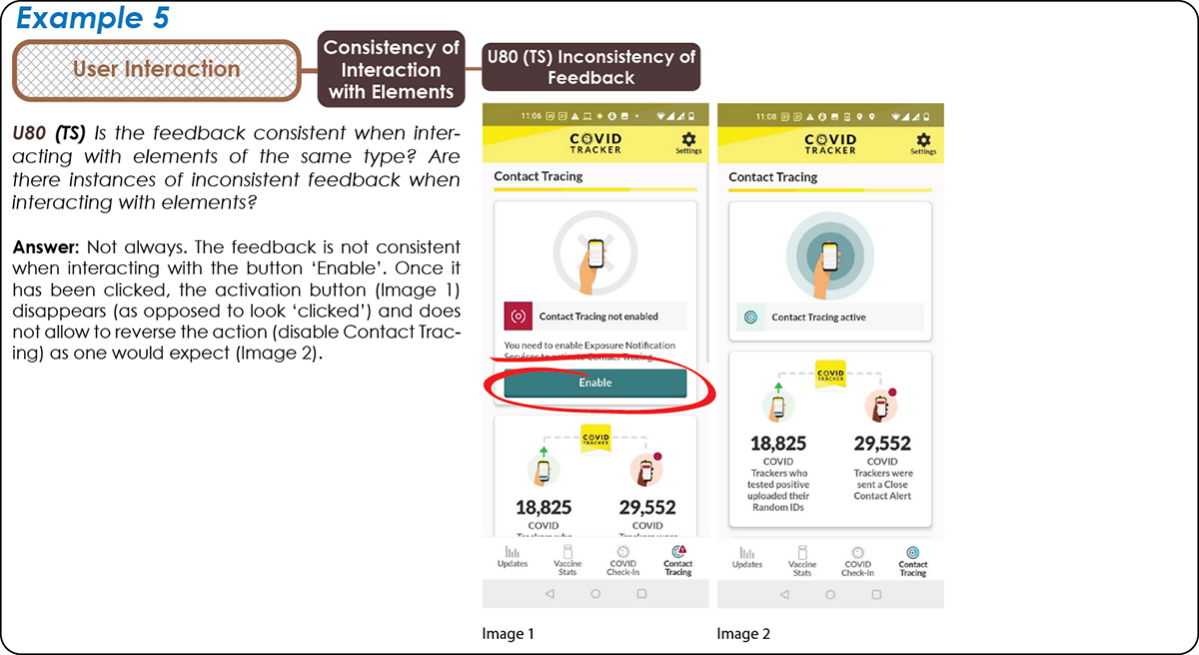
Example 5, COVID Tracker app (Ireland) [86]: inconsistency of feedback when clicking on the button “Enable”.

*User Interaction* helps to assess the user’s interaction with the app interface in the execution of specific tasks, which are identified under the above-mentioned *Completeness* attribute. Similar to *Design Effectiveness*, *User Interaction* is important to ensure correct use of the CTA and lessening user frustration; however, in contrast to design effectiveness that considers what the interface statically offers, looks, and conveys (eg, affords), user interaction considers how the interface behaves when users interact with it (eg, feedback). It includes five subattributes: *Efficiency, Robustness, Clarity of Interaction with Elements, Consistency of Interaction with Elements,* and *Alerts and Notification Messages*. Most of the selected subattributes and their related questions are *task-specific*: similar to element-specific attributes, task-specific attributes and questions are specific to users carrying out one specific task from beginning to end (eg, activating/deactivating the contact tracing functionality). Therefore, to answer task-specific questions, app inspection is needed. *Efficiency* explores the capacity of the system to produce appropriate results in return for the resources that are invested. Here, we considered three elements: Human Effort, as the number of steps that are needed to carry out a core task; Time, which is the time needed to perform that task; and the Tied-up Resources, representing the potential need for external resources (eg, power or internet) to perform the task. *Robustness* deals with the capacity of the system to adapt to different user preferences and contexts of use but also its ability to deal with user errors. With our attributes, we look at: landscape/portrait mode, multitasking when using technologies such as Bluetooth or GPS for more than one app/task (eg, while using Bluetooth earphones), and the availability of multiple ways to achieve task execution (eg, shortcuts). Adaptability looks at supporting task execution in different environments (eg, in the dark), while Errors looks at error messages available in the app as a result of inappropriate interaction and the availability of error recovery options (eg, undo, redo) or the reversibility of user actions. *Clarity of Interaction with Elements* is concerned with the clarity of what can be done with the elements available in the app interface (namely their affordances) and what happens when users interact with these elements (eg, with respect to clarity/confusion of app feedback). *Consistency of Interaction with Elements* is next, which looks at potential consistencies of Actions across the elements, the Inconsistency of Feedback, and the use of Design Constraints (if any) to prevent human errors/guide users toward correct use (Figure 2, Example 4). In our analysis, we realized the importance of feedback on the contact tracing functionalities, as we noted a number of apps, especially in their early versions, that failed to offer clear feedback to the users after the contact tracing functionality was enabled. In most cases, users had to exit the app to enable, for instance, Bluetooth, and this can create confusion for the user. In some of these cases (Figure 3, Example 5), the button enabling contact tracing did not allow reversing the action (eg, click again to disable contact tracing) that could only be reversed by disabling Bluetooth from phone settings (not from within the app), which is also problematic in terms of users felling in control of the app.

*Alerts and Notification Messages* is the last subattribute of the User Interaction attribute, which refers to the alert messages and notifications used in the app. We included attributes and questions to assess various types of Alert Messages used in the app and to assess the availability of Notification Controls, particularly for built-in notification settings in the app and for Notification Messages that alert users who have been in close contact with someone that reported a positive COVID-19 test. This part closes with a question concerning the ability of the user to access and perhaps even manipulate or visualize the generated contact tracing data (eg, number of contacts in a day, week, etc).

*Ongoing App Evaluation* refers to the app’s maintenance and upgrading, as these are important to maintain retention while also targeting new emerging needs. It includes only one subattribute looking at the Frequency of Upgrade: this can be found in Google Play and App Store, where the app can be downloaded and installed.

### Data Protection

The *Data Protection* pillar consists of two subcategories: *Security* and *GDPR*. The GDPR category focuses upon the rights of the individual citizens, while the security category takes a more data-centric view (Table 2).

*Security* consists of 4 criteria, which center around contract tracing–specific security threats and vulnerabilities. They are scoped to ensure that CTAs are compared fairly, such that security vulnerabilities related to software or system components that cannot be changed by the CTA development are not considered; for example, those related to the system security of third-party providers (third-party vendors are noted in “General Characteristics,” under “development partners,” so that an indirect warning flag is retained). These attributes incorporate a novel approach to CTA evaluation that was developed to ensure a lightweight comparison using the potentially incomplete data available for each app: analyzing vulnerabilities of distinct app functionalities against a common threat assessment model [96]. Attributes under Security (namely, STRIDE [Spoofing, Tampering, Repudiation, Information Disclosure, Denial of Service, and Elevation of Privilege] taxonomy/vulnerabilities, contact tracing–specific threats, software architecture security, and Software Development Life Cycle [SDLC] and Security) are designed to indicate whether these vulnerabilities are bugs in the code, which can be fixed or would require a redesign of the architecture to address. For example, when we used this approach to compare the security of two CTAs (Corona-Warn [89] and MyTrace [97]), we first used automated tools to identify Common Weakness Enumerators (CWEs; a categorized “encyclopedia” of over 600 types of software weaknesses), and then we manually confirmed them using in-house security expertise. We compared the identified enumerators for both apps (Table 3) and then against the predefined common threat assessment model (Table 4), providing an answer to our Security questions. Our analysis showed that while both apps may suffer from similar concerns related to information disclosure and deanonymization, the CWEs that enable these are different, with our questions under SDLC and Security allowing us to capture if the identified vulnerabilities can easily be patched/fixed or would be more difficult to correct.

*GDPR* considerations are important because they speak to the essential user concern of data privacy. The GDPR attribute has three subattributes, including Preliminaries, GDPR Principles, and Rights. Preliminaries involves information required for evaluation of the individual data stored later: Data Stored, Data Type, and Basis for Processing. It also includes Withdrawal and whether the organization declared consent and has a legal requirement as their basis for processing the data. For instance, applying question DP06 (Data Stored): “What personal data are collected?” to the COVID Tracker App [86] will generate the following list: *phone number, date of last exposure, sex, age range, county, town, symptoms, diagnosis keys, date of symptom onset, app metrics, IP address, and app security tokens*. GDPR Principles refer to the key principles of GDPR (such as Minimization, Fairness, and Storage Limitation), which are not under scrutiny in other dimensions of our C^3^EF framework. It consists of 5 attributes that are evaluated across the stored data retrieved from the question on Data Stored. Most of these criteria require details from the data controller, and must rely on those details being truthful and accurate. Our final attribute under GDPR is Rights, which refer to the rights of the individual that must be upheld if they are the subject to data processing by an organization. Therefore, they refer to the availability of organizational procedures to ensure these rights. We have 5 attributes under GDPR Rights: Access; Object to Reuse; Portability; Automated Processing Rejection; and Rectified, Restricted, or Erased (data). This is where a key distinction can be captured between CTAs that notify direct exposure to positive cases and the NOVID approach, which we discussed under Characteristics above. The way data are aggregated in NOVID creates a conflict with an individual’s right under the GDPR, as exercising a right that results in removal or change of these data (eg, the right to withdraw) will affect the wider data set. Additionally, such a data model would create conflicts with core GDPR principles such as data minimization, and avoidance of user may result in reidentification, through the combination of multiple data sources. Similarly, the AMAN app [90], which is not based in an EU state and, as such, is only required to adhere to the GDPR if it is used by EU citizens, had a distinct lack of documentation to support GDPR rights, as is to be expected.

**Table 3:** Comparison of Common Weakness Enumerators (CWEs) in the Corona-Warn [89] and MyTrace [97] apps.

CWE	Corona-Warn	MyTrace
89: A (SQL^a^) Command	Local SQL injection possible but data encrypted	Local SQL injection possible and data not encrypted
276: Incorrect Default Permissions	N/A^b^	Permissions for tasks, Bluetooth administration, and external storage
295: Improper Certificate Validation	Vulnerable to SSL^c^ MITM^d^ attack	N/A
532: Insertion of Sensitive Information into Log File	Sensitive information is encrypted	Excessive information logged
327: Use of a Broken or Risky Cryptographic Algorithm	Weak hash function in SSL	N/A

^a^SQL: Structured Query Language.

^b^N/A: not applicable.

^c^SSL: Secure Socket Layer.

^d^MITM: man in the middle.

**Table 4:** Comparison of threats to Corona-Warn [89] and MyTrace [97] using the Common Weakness Enumerators (CWEs) listed in Table 3 (with a severity rating: H=high, M=medium, and L=low) against the common threat assessment model.

Threat	Corona-Warn Matched CWEs	MyTrace Matched CWEs
Fake alert injection	N/A^a^	CWE-327-H
False report	CWE-295-H, CWE-327-L	N/A
Proximity beacons altered	N/A	CWE-89-H
User can deny or retract infection report or contact details	CWE-295-H	N/A
Personal information disclosed	CWE-327-L, CWE295-H	CWE-89-H, CWE-276-H
User deanonymized and tracked	CWE327-L, CWE295-H	CWE-89-H, CWE-276-H, CWE-532-H
Energy resource drain attack	N/A	CWE-276-H
System resource contention	N/A	CWE276-H

^a^N/A: not applicable.

### Effectiveness

The *Effectiveness* pillar refers to the degree to which the app is successful in its core aims: accurately detecting close contacts and thus providing “notification to other app users with potential exposure risks to an infected app user” [98]. It contains three high-level attributes (see Table 2), the first of which (*Effective Reporting*) refers to concerns related to accurate detection, and the second of which (*Effective Results*) refers to providing notification to other app users with potential exposure risks, a concern provisionally referred to as “performance” by other commentators in the field [77]. The third attribute (*Effective Engagement*) refers to the “other app users” and “infected app users” in the definition, specifically focusing on the level of app adoption by citizens.

**Figure 4:**
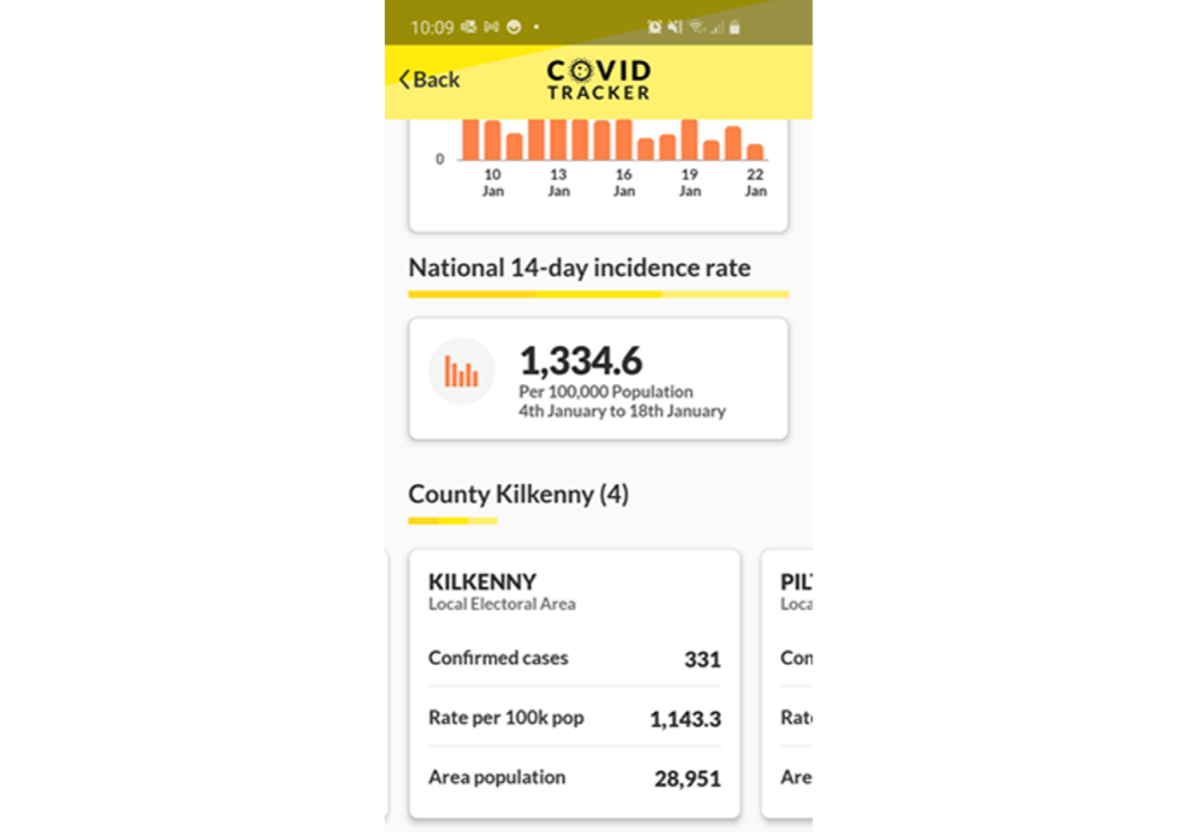
Electoral district–level COVID-19 statistics on the Health Service Executive's COVID Tracker app (Ireland) [86].

*Effective Reporting* focuses on the ability of the app to report accurately on close contacts and the location of virus hotspots to individual users. It first assesses the accuracy of close contact detection: often, this will have to be reported at the protocol level, for example, stating that the app reports at GAEN-level accuracy [78]. The framework then breaks down the reporting of close contacts into two categories: reporting on COVID-19–positive close contacts and reporting on a user’s total number of close contacts over time periods. This latter category is sometimes reported in an effort to highlight and refine users’ behavior, as in the case of PathCheck SafePlaces [87]. Finally, several of the apps report on prevalence information by locale to let users know areas where the virus is more (or less) prevalent. This is a form of hotspot identification for users (see Figure 4 for an example from the HSE’s COVID Tracker app [86]). The final question in this section probes the availability/granularity of this hotspot facility (“electoral division” in the case of Figure 4).

*Effective Results* focuses on the ability of the app to meet its wider objectives across the jurisdiction. Hence, it concentrates on the (total) number of users who choose to share their data after being told of their positive status and the number of additional close contacts that are informed, based on that sharing. It then looks at the number of those contacts who were subsequently identified as positive. Finally, it aims to assess the relative time and effort in identifying a close contact via the app, as opposed to via the manual-tracing effort.

*Effective Engagement* focuses on the population’s adoption of the app, probing the uptake of the app across the population; citizens’ retention of the app over time; and, in cases where the app contains interactive features, the population’s engagement with the app, as possibly measured by their usage of these interactive features. This is important because digital contact tracing is very dependent on the proportion of the population who upload the associated app and retain it over time.

### Transparency

Transparency is discussed here as an independent pillar, despite the obvious overlap with GDPR. This is because here it addresses the transparency of the processes and artifacts utilized/formed during development of the app specifically. In this context, transparency is an important aspect from the perspective of adopting citizens’ confidence. The pillar has been divided into categories looking at *App Transparency, User Participation,* and *Data Transparency* (Table 2).

*App Transparency* includes App Purpose and App Permission. App Purpose offers the attributes (1) App-Purpose Knowledge, which refers to the purpose of the app being made accurately and accessibly explicit to the adopting citizen; (2) App Participation Knowledge, which looks at whether the citizens receive a clear explanation of the voluntary nature of participation; (3) App Development Knowledge, which looks at the mechanisms employed to guarantee community feedback; and (4) Open Source Repository to assess if the source code is made available (eg, on GitHub), as this too shows transparency at a high level. App Permission investigates all the permissions that are being asked for by the app, such as permission to access Bluetooth/the camera, in terms of how transparent the app is about these phone functionalities accessed. Modus Operandi probes the CTA’s transparency regarding the permissions required for its functionality, and looks at the time period over which the services are being used as well as the contact tracing accuracy claimed by the developers (eg, with questions such as “Is the app being transparent about the contact tracing accuracy that they are achieving?”).

*User Participation* consists of only one question, ensuring user consent: “Does the app indicate and explain to the end user about the voluntary nature of participation?”

*Data Transparency* focuses on whether the app has been designed following a privacy-by-design principle (under Data Capture Knowledge), as this will heighten confidence as to its data privacy nature. It assesses if the users are made accurately and accessibly aware of the data accessible to other bodies, both in terms of the data and the accessing bodies (Data-Access Knowledge). It probes if the users are made explicitly aware of where and for how long their data are stored. “Privacy policy knowledge” is also of concern here, looking at if, how, and when the citizen is informed about the data being collected in a Data Protection Impact Assessment. Two more attributes look at the “minimality” of the collected personal data and at Data Protection, which focuses exclusively on the transparency section of data encryption and data anonymity. It has several questions that assess the anonymity, encryption protocol, and the end-of-life conditions for the data.

### Technical Performance

Technical Performance defines how efficiently a software system operates (in contrast to effectiveness), and it includes attributes and questions that help to capture this operational efficiency. The main attributes populating the Technical Performance pillar are: *Speed, Efficiency, Consumption,* and *Resource/Troubleshooting and Trust*.

The *Speed* subattribute captures how quickly a software system’s frontend app responds to a user’s requests, as delays may cause user frustration. This subattribute probes two issues. The first queries how fast the app responds to a user’s interaction. The response here is measured in time units (eg, milliseconds), and can be influenced by several aspects such as third-party apps (including an operating system’s libraries and API), hardware and its configurations, various components of the app, and how they work together.

The second *Efficiency* question focuses on the algorithms of the app that are responsible for answering a user’s requests.

*Consumption* and *Resources/Troubleshooting and Trust* capture how efficiently a software system consumes available hardware resources, including efficiency of battery usage, disk usage, CPU and memory usage, and bandwidth consumption. These attributes are particularly important with respect to retention if users perceive a battery/storage/CPU drain on their device [79] and so should probably be assessed as above or below according to some sort of “noticeable-threshold” level.

### Citizen Autonomy

The Citizen Autonomy pillar refers to the degree to which a user has the ability to define their own control levels in terms of the rights and accesses they grant the app. Additionally, it is concerned with the user’s ability to influence the evolution of the app going forward: an important element of autonomy, given that jurisdictions are asking users to retain the app for the duration of the emergency.

The pillar has three high-level attributes: *App Discussion Authority, Phone Functionality*, and *Data Control* (see Table 2). Cumulatively, these three categories consist of 9 questions.

**Figure 5:**
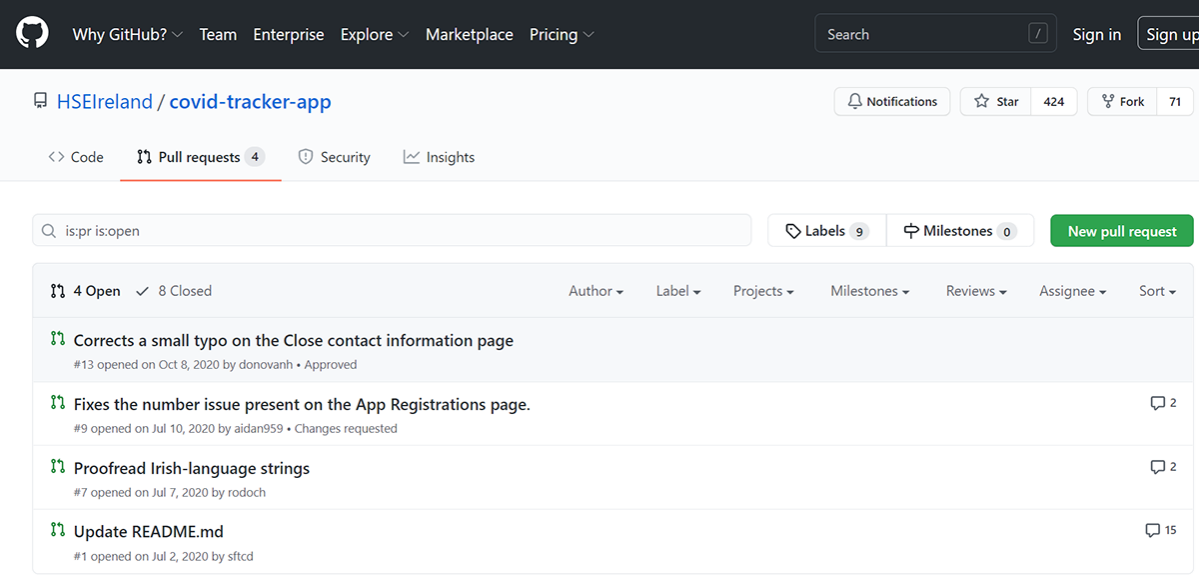
The "pull requests" GitHub page [99] for the Health Service Executive's COVID Tracker app [86].

*App Discussion Authority* focuses on the ability of the user to influence the future direction of the app and thus feel a sense of ownership. It first checks if there is a discussion forum where users are free to leave opinions and requests for change (most apps have at least a review section on Google Play or Apple’s App Store by default). An important consideration then is whether the available review sites are curated or moderated by representatives of the app development team. For example, the HSE’s COVID Tracker app [86] has reviews on Google Play and Apple Store, but it also has a GitHub repository [99] where users can leave their push requests for developers, as illustrated in Figure 5.

*Phone Functionality* focuses on the ability of the user to control the app’s access to phone services. Typically, these are relevant services such as GPS, GAEN [78], Bluetooth, and notifications, where their role in the app’s functioning is apparent. However, occasionally, additional services may be required. For example, NOVID [88] uses ultrasound in an attempt to make close-contact detection more accurate and, as a result, requires access to the phone’s microphone.

*Data Control* focuses primarily on the data that are uploaded from the user’s app, typically in an instance of a positive diagnosis for the virus. It checks if the user is explicitly asked their permission for the upload of these sensitive data to happen and if the user is made aware of where the resultant data are stored (and by whom).

## Discussion

### Significance of the Framework

The research question of this study revolved around devising and organizing a framework to enable a more comprehensive assessment of current CTAs, thus supporting the work of decision-makers (eg, developers, health authorities) in the development and evolution of CTAs, and potentially increasing citizens’ adoption. While evaluation frameworks exist, they tend to focus on specific aspects of CTAs, and we wanted to enable a more holistic assessment that could help to compare and improve the design of CTAs across a series of dimensions important for citizen adoption. For this purpose, we also wanted to assess what is available in the literature and ground what we derived with empirical and iterative tests run using a selection of CTAs. In doing this, we formulated and added probing questions (and sample answers) to the attributes derived for our framework, which we consider another key contribution, in terms of its application by interested stakeholders.

### Open-Ended Nature of C^3^EF

Questions in C^3^EF were mainly formulated to assess the essential functionality of the CTAs (ie, the contact-tracing function). Nonetheless, one important consideration at this stage concerns the open-ended nature of C^3^EF. Their iterative derivation and refinement make particularly apparent how CTAs are constantly changing and evolving. Not only are CTAs (more or less) frequently updated to fix issues and improve functionalities, but they also operate in a changing scenario, which offers new requirements and design opportunities over time. For instance, at the beginning of our project, vaccines were not available. However, at the time of writing, a number of vaccines are being administered, which opened up new needs (digital vaccine passports, interoperability between systems when traveling abroad) that might extend the scope of current CTAs as we know and evaluate them now. We do not see this as a limitation of our work, but rather as an invitation to progress research on this topic and to extend the framework. Moreover, pillars such as Usability, Data Protection, Citizen Autonomy, and Transparency offer several questions that are agnostic to the types of functionalities under scrutiny, and can be easily applied to nonessential functionalities such as check-in functions, statistic dashboards, displaying tests, and vaccine certificates, among others. In this sense, our framework can support assessment of mHealth apps that are not necessarily focused on contact tracing, although a number of questions remain specific to CTAs and are not necessarily applicable outside this domain.

Reporting transparently on our methodology, while also sharing a supporting website that offers interested stakeholders public access to the framework and the ability to send us feedback [93], represent strategies to facilitate adoption of our framework and adapting it to new emerging questions and needs. In this sense, we hope that researchers will engage, criticize, and improve on those attributes to support improved and expanded CTAs over time, but we also hope that stakeholders can use the included evaluation concerns as guidance when designing, evolving, or evaluating CTAs.

### Use of the Framework

The examples we share in our Results section (Figures 2-5, Tables 3 and 4) are meant to illustrate how the framework can be used to assess or compare apps. The adding of attribute-related questions, sample answers from our own tests, visual representations of the structure of our taxonomy, and, once again, a website that allows others to use the framework in the form of a survey (with the opportunity to leave feedback on the application of the framework) are all devices that we devised to support potential stakeholders (eg, developers and health authorities) in using our framework to develop, evolve, and improve the design of CTAs with an eye on citizen adoption.

Although the questions tend to be descriptive in nature, the framework can also be used as a checklist of important elements to be considered. In this sense, the framework is prescriptive, as it helps developers appreciate either desirable qualities (eg, asking if there is a forum for citizens’ feedback also implies that it would be desirable for CTAs to offer such a forum to citizens). Likewise, it can inform them of design tradeoffs. For example, while more functionalities and actionable information might make the CTAs more attractive, these might also make them more difficult to use. Another example is the alternative (CTA) approach that warns users in a social network *prior* to exposure to a positive case (as implemented in NOVID). This could be more appealing to citizens, but it is potentially more vulnerable to data deanonymization.

Most of our questions are devised to be answered via app inspection, inspection of the app website, or inspection of the page in the app stores (eg, Characteristic, Transparency, Usability, and Citizens’ Autonomy pillars). We assume that interested stakeholders possess the required skills to independently answer the questions of these pillars. In some other cases, answering questions could be more difficult in terms of feasibility but also in terms of required competences. For example, the security section of the Data Protection pillar analyzes vulnerabilities of distinct app functionalities against a common threat assessment model [96], and this requires technical skills and familiarity with system security tools. Similarly, Technical Performance assessment assumes a user with a background in software engineering.

In terms of feasibility, we often noted a tension between the importance of an attribute and the feasibility of getting the information to answer the attribute’s question. A good example is presented under Effectiveness, where the number of users tested early in response to an app notification is considered a core measure. However, this is difficult to ascertain without significant buy-in from and integration with the national health authority working to capture and make such data available. However, we believe that the framework should ultimately contain important attributes, even when it is difficult to obtain an answer, as they often highlight the core criteria that should be used to assess the apps, and thus reflect a need for wider buy-in by the associated health authority and/or citizens.

### General CTA Design Considerations

As part of our derivation and consolidation phase, we had to apply our framework (its early, iterated, and finalized versions) to the 5 apps, and this put us in a position to appreciate a number of issues we found across all CTAs and that further confirm the ability of our framework to capture critical aspects. For instance, some of the questions to assess Universality and Accessibility (under Usability) revealed that most apps do not support minors as users under parental/legal guardian consent, and that interfaces and functions do not seem to be designed to accommodate use by children under the age of 13 years. We consider this to be an issue given that the vaccination campaign for individuals under 13 is still not clear and they are a large cohort in most national populations.

As mentioned above, we also found that most apps lack clear and actionable feedback, which can sometimes be conducive to confusion (eg, is my contact tracing active?) and lack of sense of control. Similarly, we found an overall scarcity of information push and synchronous interactive features, including that of synchronous assistance (only offered by Corona-Warn in Germany). This lack of synchronous, interactive features has the potential to negatively affect adoption because the perceived benefits of CTAs are not made sufficiently apparent. In a related work, we argued how feedback and providing more diverse and actionable information to citizens (eg, number of daily contacts, proximity buzz, hotspots) was seen as key to maintain engagement in CTAs.

Finally, we also note that, in terms of security, individual CWE issues are not standard across CTAs. Even so, individual security issues seem to arise in CTAs, based on individual CWE issues specific to the CTAs reviewed. Exacerbating this concern is the fact that the framework does not assess the complete CTA, ignoring third-party components with respect to security.

### Conclusions

Our stated research question was “how to devise and organize a framework to enable a more comprehensive assessment of current CTAs, supporting the work of decision-makers (eg, developers, health authorities) in the development and evolution of CTAs, potentially increasing adoption?” While the “how” part of this question is largely addressed in our Methods section, the resultant citizen-focused CTA framework provides a holistic compare-and-contrast tool that supports the work of decision-makers in this sphere. It aims to support reflection on developers’ design decisions so as to better understand and optimize the design compromises in play. As such, we see it as a vital tool for designers designing and evolving current CTAs for increased public adoption. However, it can also be used by commentators in the assessment of CTAs, more generally, across jurisdictions to identify more optimal alternatives and prevalent, problematic issues. Such commentaries can be an important tool when governments are looking for CTA solutions to adopt or mimic.

For these purposes, it is important that we continue to assess existing and new CTAs against the framework, and document the results. As a prerequisite, we must also develop “best-practice” guidelines for performing app evaluations when using the framework. Both of these agendas are areas of ongoing concern for us in the “COVIGILANT” project. Ideally, this work will result in an openly accessible protocol and a database of framework application results, where CTA designers/developers will also have the right to reply (to heighten traceability), and these facilities would act as a single resource where designers could go to obtain a broad comparison over the CTAs available.

Nevertheless, there are still issues to address: the example discussed earlier assumes, for instance, that data about the number of citizens alerted of their COVID-19 positivity and the number who decide to get tested because of a CTA notification exist, and that these numbers are monitored. This is only possible if certain procedures are established by health authorities (such as keeping records of individuals who ask for a test because of a notification from their CTAs). Our framework focuses on the app itself; however, to have a significant impact on the spread of the virus, CTAs need to be integrated as part of the pandemic response through an ecosystem of organizational, political, and social entities, which goes well beyond the scope of our framework. The temporary failure of health systems in dealing with digital contact tracing has been well documented [21], and this means that *scoring well* on our C^3^EF does not necessarily guarantee that the CTAs will have an evaluable impact on containing the spread of the virus. 

Another limitation is that target users were not directly involved in the initial derivation of our C^3^EF. The project was run during the first lockdown in Europe and, despite the team having connections with CTA developers in Ireland, the Irish health authorities, and the European CDC, we acknowledge that the interactions held with these bodies were informal sessions toward the end of the derivation process. In those sessions, they provided feedback to us on the framework and, in the case of the CDC, we contributed to their effectiveness framework [100] using the insights obtained to identify small refinements in our own framework. We desisted from detailing these interactions in our Methods section based on their lack of formality, and acknowledge that more should have been done to incorporate these central stakeholders in the derivation. To partially address this, we have made our framework publicly available and offer the opportunity for users to leave feedback in an attempt to support future engagement with stakeholders. We appreciate that this is unlikely to happen without further directed work, and this too is an area of future work for us.

A final potential limitation of this work is the methodology employed. While the differing approaches employed can be considered a form of data triangulation, and thus a positive attribute [101,102], the data were nonindependent, with earlier data being used as the foundation for later data generation/refinement. In addition, the protocol we used for the first part of phase 2 (applying the provisional framework to 5 CTAs) and phase 3 of the analysis (Refinement and Finalization) were derived specifically for this study, and not based on any established method. However, this was because of the specific goals of these analysis phases: determining relevancy, sufficiency, specificity, and redundancy issues in the framework. In addition, the extensive, immersive, and targeted nature of the application/refinement sessions, along with the final evaluation of the resultant framework against existing CTAs suggest a level of rigor in these phases that is defensible.

## Appendix

Multimedia Appendix 1 A summary of all papers identified for the literature review. Initial list of 111 extracted attributes and categories, our analysis, and grouping into 6 categories.

Multimedia Appendix 2 Snippet of the cross-pillar/ambiguity analysis matrix.

Multimedia Appendix 3 Visualization of all pillars and questions.

## Abbreviations

API: application programming interface
C^3^EF: Citizen-Focused Compare-and-Contrast Evaluation Framework
CDC: European Centre for Disease Control
CTA: contact tracing app
CWE: Common Weakness Enumerator
DP-3T: Decentralized Privacy Preserving Proximity Tracing
GAEN: Google and Apple Exposure Notification
GDPR: General Data Protection Regulations
HSE: Health Service Executive (Ireland)
mHealth: mobile health
MIT: Massachusetts Institute of Technology
SDLC: Software Development Life Cycle
STRIDE: Spoofing, Tampering, Repudiation, Information Disclosure, Denial of Service, and Elevation of Privilege
UD: Universal Design

## References

[1] Baxter S, Goyder E, Chambers D, Johnson M, Preston L, Booth A (2017). Interventions to improve contact tracing for tuberculosis in specific groups and in wider populations: an evidence synthesis. Health Serv Deliv Res.

[2] Swanson KC, Altare C, Wesseh CS, Nyenswah T, Ahmed T, Eyal N, Hamblion EL, Lessler J, Peters DH, Altmann M (2018). Contact tracing performance during the Ebola epidemic in Liberia, 2014-2015. PLoS Negl Trop Dis.

[3] Anglemyer A, Moore TH, Parker L, Chambers T, Grady A, Chiu K, Parry M, Wilczynska M, Flemyng E, Bero L (2020). Digital contact tracing technologies in epidemics: a rapid review. Cochrane Database Syst Rev.

[4] Danquah LO, Hasham N, MacFarlane M, Conteh FE, Momoh F, Tedesco AA, Jambai A, Ross DA, Weiss HA (2019). Use of a mobile application for Ebola contact tracing and monitoring in northern Sierra Leone: a proof-of-concept study. BMC Infect Dis.

[5] Ming LC, Untong N, Aliudin NA, Osili N, Kifli N, Tan CS, Goh KW, Ng PW, Al-Worafi YM, Lee KS, Goh HP (2020). Mobile health apps on COVID-19 launched in the early days of the pandemic: content analysis and review. JMIR Mhealth Uhealth.

[6] eHealth Network (2020). Mobile applications to support contact tracing in the EU's fight against COVID-19: Common EU Toolbox for Member States. European Comission.

[7] COVID-19 Contact tracing: data protection expectations on app development. Information Commission Office (ICO).

[8] Parker MJ, Fraser C, Abeler-Dörner L, Bonsall D (2020). Ethics of instantaneous contact tracing using mobile phone apps in the control of the COVID-19 pandemic. J Med Ethics.

[9] (2020). Digital tools for COVID-19 contact tracing. World Health Organization.

[10] Jacob S, Lawarée J (2020). The adoption of contact tracing applications of COVID-19 by European governments. Policy Design Pract.

[11] Walrave M, Waeterloos C, Ponnet K (2020). Adoption of a contact tracing app for containing COVID-19: a health belief model approach. JMIR Public Health Surveill.

[12] Martin T, Karopoulos G, Hernández-Ramos JL, Kambourakis G, Nai Fovino I (2020). Demystifying COVID-19 digital contact tracing: a survey on frameworks and mobile apps. Wirel Commun Mob Comput.

[13] Vinuesa R, Theodorou A, Battaglini M, Dignum V (2020). A socio-technical framework for digital contact tracing. Results Eng.

[14] Cho H, Ippolito D, Yu Y (2020). Contact Tracing Mobile Apps for COVID-19: Privacy Considerations and Related Trade-offs. arXiv.

[15] De Carli A, Franco M, Gassmann A, Killer C, Rodrigues B, Scheid E, Schoenbaechler D, Stiller B (2020). WeTrace -- A privacy-preserving mobile COVID-19 tracing approach and application. arXiv.

[16] Gasser U, Ienca M, Scheibner J, Sleigh J, Vayena E (2020). Digital tools against COVID-19: Framing the ethical challenges and how to address them. arXiv.

[17] Dar AB, Lone AH, Zahoor S, Khan AA, Naaz R (2020). Applicability of mobile contact tracing in fighting pandemic (COVID-19): issues, challenges and solutions. Comput Sci Rev.

[18] Bente BE, van 't Klooster JWJR, Schreijer MA, Berkemeier L, van Gend JE, Slijkhuis PJH, Kelders SM, van Gemert-Pijnen JEWC (2021). The Dutch COVID-19 contact tracing app (the CoronaMelder): usability study. JMIR Form Res.

[19] Weiß JP, Esdar M, Hübner U (2021). Analyzing the essential attributes of nationally issued COVID-19 contact tracing apps: open-source intelligence approach and content analysis. JMIR Mhealth Uhealth.

[20] (2021). Europe for Privacy-Preserving Pandemic Protection (E4P); Comparison of existing pandemic contact tracing systems. ETSI.

[21] von Wyl V (2021). Challenges for nontechnical implementation of digital proximity tracing during the COVID-19 pandemic: media analysis of the SwissCovid app. JMIR Mhealth Uhealth.

[22] Saunders M, Lewis P, Thornhill A (2019). Research Methods For Business Students. 8th ed.

[23] Wohlin C (2014). Guidelines for snowballing in systematic literature studies and a replication in software engineering.

[24] Morley J, Cowls J, Taddeo M, Floridi L, Cowls J, Morley J (2021). Ethical guidelines for SARS-CoV-2 digital tracking and tracing systems. The 2020 Yearbook of the Digital Ethics Lab. Digital Ethics Lab Yearbook.

[25] Lodders A, Paterson JM (2020). Scrutinising COVIDSafe: frameworks for evaluating digital contact tracing technologies. Alt Law J.

[26] Sanwikarja P (2020). Contact tracing: How do you design an app millions of people will trust?. UX Collective.

[27] Kasali F, Taiwo O, Akinyemi I, Alaba O, Awodele O, Kuyoro S (2019). An enhanced usability model for mobile health application. Int J Comput Sci Inf Security.

[28] van Haasteren A, Gille F, Fadda M, Vayena E (2019). Development of the mHealth app trustworthiness checklist. Digit Health.

[29] Harrington C, Ruzic L, Sanford J, Antona M, Stephanidis C (2017). Universally accessible mHealth apps for older adults: towards increasing adoption and sustained engagement. Universal Access in Human–Computer Interaction. Human and Technological Environments. UAHCI 2017. Lecture Notes in Computer Science, vol 10279.

[30] (2018). Accessibility requirements for ICT products and services. EN 301 549 V2.1.2. ETSI.

[31] (2019). Xcertia mHealth App Guidelines. Healthcare Information and Management Systems Society (HIMSS).

[32] García-Vidal J Interoperable Digital Proximity Tracing Protocol (IDPT). UP Commons.

[33] Rossignol J (2020). Apple and Google reveal how COVID-19 exposure notification apps will function. MacRumors.

[34] Clover J (2021). Apple's exposure notification system: everything you need to know. MacRumors.

[35] (2020). Principles for legislators on the implementation of new technologies. Irish Council for Civil Liberties (ICCL).

[36] HSE Covid Tracker App: Pre-Release Report Card. Irish Council for Civil Liberties (ICCL).

[37] Mobile applications to support contact tracing in the EU's fight against COVID-19. Progress reporting June 2020. European Commission.

[38] COVID-19 exposure notification using bluetooth low energy. Apple/Google.

[39] Lillington K Use Covid tracker app if you wish, but be sure to wear a face covering. The Irish Times.

[40] Criddle C, Kelion L Coronavirus contact-tracing: World split between two types of app - BBC News. BBC.

[41] Moss A, Spelliscy C, Borthwick J (2020). Demonstrating 15 contact tracing and other tools built to mitigate the impact of COVID-19. TechCrunch.

[42] (2020). COVID-19 contact tracing apps: Here is how different countries are taking different approaches. Firstpost.

[43] O'Neill P, Ryan-Mosley T, Johnson B A flood of coronavirus apps are tracking us. Now it's time to keep track of them. MIT Technology Review.

[44] Kaur E, Haghighi P (2016). A context-aware usability model for mobile health applications.

[45] Kascak L, Rebola C, Sanford J (2014). Integrating Universal Design (UD) principles and mobile design guidelines to improve design of mobile health applications for older adults.

[46] Ballantyne M, Jha A, Jacobsen A, Scott HJ, El-Glaly Y (2018). Study of accessibility guidelines of mobile applications.

[47] Alturki R, Gay V, Jan M, Khan F, Alam M (2019). Usability attributes for mobile applications: a systematic review. Recent Trends and Advances in Wireless and IoT-enabled Networks. EAI/Springer Innovations in Communication and Computing.

[48] Goel S, Nagpal R, Mehrotra D, Mishra D, Nayak M, Joshi A (2018). Mobile applications usability parameters: taking an insight view. Information and Communication Technology for Sustainable Development. Lecture Notes in Networks and Systems, vol 9.

[49] Shitkova M, Holler J, Heide T, Clever N, Becker J (2015). Towards usability guidelines for mobile websites and applications.

[50] Weichbroth P (2020). Usability of mobile applications: a systematic literature study. IEEE Access.

[51] Alonso-Ríos D, Vázquez-García A, Mosqueira-Rey E, Moret-Bonillo V (2010). Usability: a critical analysis and a taxonomy. Int J Hum-Comput Interact.

[52] Soni N, Aloba A, Morga K, Wisniewski P, Anthony L (2019). A framework of Touchscreen interaction design recommendations for children (TIDRC): Characterizing the gap between research evidence and design practice.

[53] Gelman D (2014). Design for kids: digital products for playing and learning. 1st ed.

[54] Art. 8 GDPR - Conditions applicable to child's consent in relation to information society services. Intersoft Consulting.

[55] Directive (EU) 2016/2102 of the European Parliament and of the Council of 26 October 2016 on the accessibility of the websites and mobile applications of public sector bodies. Eur-Lex.

[56] Web Content Accessibility Guidelines (WCAG) Overview. Web Accessibility Initiative (WAI), W3C.

[57] Silver A (2016). Improving The Color Accessibility For Color-Blind Users. Smashing Magazine.

[58] Vokinger KN, Nittas V, Witt CM, Fabrikant SI, von Wyl V (2020). Digital health and the COVID-19 epidemic: an assessment framework for apps from an epidemiological and legal perspective. Swiss Med Wkly.

[59] Lo B, Sim I (2021). Ethical framework for assessing manual and digital contact tracing for COVID-19. Ann Intern Med.

[60] Righi C, James J, Beasley M, Day D, Fox J, Gieber J, Howe C, Ruby L (2013). Card sort analysis best practices. J Usability Stud.

[61] Nielsen J (1993). Usability engineering.

[62] Norman D (2013). The design of everyday things: revised and expanded edition. 2nd edition.

[63] Preece J, Sharp H, Rogers Y (2015). Interaction design: beyond human-computer interaction. 4th edition.

[64] Storni C, Tsvyatkova D, Richardson I, Buckley J, Abbas M, Beecham S, Chochlov M, Fitzgerald B, Glynn L, Johnson K, Laffey J, Mcnicholas B, Nuseibeh B, Connell J, Keeffe D, Keeffe I, Callaghan M, Razzaq A, Rekanar K, Simpkin A, Walsh J, Welsh T (2021). Toward a compare and contrast framework for COVID-19 contact tracing mobile applications: a look at usability.

[65] Ayala-Rivera V, Pasquale L (2018). The grace period has ended: An approach to operationalize GDPR requirements.

[66] General Data Protection Regulation (GDPR). Intersoft Consulting.

[67] Know Your Obligations. Data Protection Commission.

[68] Guide to the UK General Data Protection Regulation (UK GDPR). Information Commissioner's Office (ICO).

[69] Horák M, Stupka V, Husák M (2019). GDPR compliance in cybersecurity software: a case study of DPIA in information sharing platform.

[70] Rios E, Iturbe E, Larrucea X, Rak M, Mallouli W, Dominiak J, Muntés V, Matthews P, Gonzalez L (2019). Service level agreement‐based GDPR compliance and security assurance in(multi)Cloud‐based systems. IET Softw.

[71] Torre D, Soltana G, Sabetzadeh M, Briand L, Auffinger Y, Goes P (2019). Using models to enable compliance checking against the GDPR: an experience report.

[72] Robol M, Salnitri M, Giorgini P, Poels G, Gailly F, Serral Asenio E, Snoeck M (2017). Toward GDPR-compliant socio-technical systems: modeling language reasoning framework. The practice of enterprise modeling.

[73] Tom J, Sing E, Matulevičius R, Zdravkovic J, Grabis J, Nurcan S, Stirna J (2018). Conceptual representation of the GDPR: model and application directions. Perspectives in business informatics research.

[74] Allegue S, Rhahla M, Abdellatif T, Yangui S (2020). Toward GDPR compliance in IoT systems. Service-Oriented Computing – ICSOC 2019 Workshops. ICSOC 2019. Lecture Notes in Computer Science, vol 12019.

[75] Tamburri D (2020). Design principles for the General Data Protection Regulation (GDPR): a formal concept analysis and its evaluation. Inf Syst.

[76] Kammueller F (2018). Formal modeling and analysis of data protection for GDPR compliance of IoT healthcare systems.

[77] Lueks W, Benzler J, Bogdanov D, Kirchner G, Lucas R, Oliveira R, Preneel B, Salathé Marcel, Troncoso C, von Wyl V (2021). Toward a common performance and effectiveness terminology for digital proximity tracing applications. Front Digit Health.

[78] Exposure notifications: using technology to help public health authorities fight COVID‑19. Google.

[79] Rekanar K, O'Keeffe IR, Buckley S, Abbas M, Beecham S, Chochlov M, Fitzgerald B, Glynn L, Johnson K, Laffey J, McNicholas B, Nuseibeh B, O'Connell J, O'Keeffe D, O'Callaghan M, Razzaq A, Richardson I, Simpkin A, Storni C, Tsvyatkova D, Walsh J, Welsh T, Buckley J (2022). Sentiment analysis of user feedback on the HSE's Covid-19 contact tracing app. Ir J Med Sci.

[80] SwissCovid. Google Play.

[81] Apturi Covid. Ministry of Health and Centre for Disease Prevention and Control.

[82] Immuni. Government of Italy.

[83] Troncoso C, Payer M, Hubaux J, Salathé M, Larus J, Bugnion E, Lueks W, Stadler T, Pyrgelis A, Antonioli D, Barman L, Chatel S, Paterson K, Capkun S, Basin D, Beutel J, Jackson D, Preneel B, Smart N, Singelee D, Abidin A, Gürses S, Veale M, Cremers C, Backes M, Binns R, Cattuto C, Persiano G, Fiore D, Barbosa M, Boneh D DP-3T documents: Decentralized Privacy-Preserving Proximity Tracing. GitHub.

[84] Decentralized privacy-preserving proximity tracing. Wikipedia.

[85] COVID-19 apps. Wikipedia.

[86] Covid Tracker App. Ireland Health Service Executive (HSE).

[87] COVID Safe Paths. PathCheck Foundation.

[88] NOVID.

[89] (2020). Corona-Warn-App Open Source Project.

[90] (2020). Aman.

[91] Safe Paths. MIT Media Lab.

[92] Kasi N (2018). Implications of an assigned devil's advocate role in a negotiations context. Thesis. Penn Libraries.

[93] Digital Contact Tracing Taxonomy.

[94] Buckley J, Storni C The challenge in striking the right balance with Covid apps. RTE.

[95] User interface elements. Usability.gov.

[96] Welsh T, Rekanar K, Abbas M, Chochlov M, Fitzgerald B, Glynn L, Johnson K, Laffey J, McNicholas B, Nuseibeh B, O?Connell J, O?Keeffe D, O?Keeffe I, O?Callaghan M, Razzaq A, Richardson I, Simpkin A, Storni C, Tsvyatkova D, Walsh J, Buckley J (2020). Towards a taxonomy for evaluating societal concerns of contact tracing Apps.

[97] MyTrace, a Preventive Counter Measure and Contact Tracing Application for COVID-19. Malaysia Ministry of Science and Technological Innovation.

[98] (2020). Policy briefs. Swiss National COVID-19 Science Task Force.

[99] (2020). HSEIreland covid-tracker-app. GitHub.

[100] (2021). Indicator framework to evaluate the public health effectiveness of digital proximity tracing solutions.

[101] Regmi K (2014). Triangulation in healthcare research: what does it achieve?. SAGE Research Methods Cases Part 1.

[102] Rutherford GW, McFarland W, Spindler H, White K, Patel SV, Aberle-Grasse J, Sabin K, Smith N, Taché S, Calleja-Garcia JM, Stoneburner RL (2010). Public health triangulation: approach and application to synthesizing data to understand national and local HIV epidemics. BMC Public Health.

