# Supplementary material for: Digital Contact Tracing Apps for COVID-19: Development of a Citizen-Centered Evaluation Framework
Source: JMIR Mhealth Uhealth. 2022 Mar 11;10(3):e30691. doi: 10.2196/30691 (PMC8919989; doi:10.2196/30691)
Supplement: Multimedia Appendix 1 [file mhealth_v10i3e30691_app1.docx]

## Multimedia Appendix 1

Table 1. A summary of all papers identified in the literature review.

| **DCTAs/**  **Mobile applications** | **Frameworks/**  **guidelines**  **/regulations** | **Aim** | **Ref.** | **Source** |
| --- | --- | --- | --- | --- |
| **1 DCTAs** | Frameworks for DCTAs | Suggest an assessment framework for DCTAs based on an existing trustworthiness checklist for mHealth applications. The framework is primarily focused on security, privacy and publisher reputation characteristics and less focused on Usability aspects. | [58] | Academic Literature |
|  |  | Suggest an ethical framework for assessing DCTAs. | [59] | Academic Literature |
|  |  | Propose WeTrace approach that considers a full privacy-preserving contact tracking and tracing. | [15] | Academic Literature |
|  |  | Discuss privacy issues and strategies for adoption of the DCTAs. | [14] | Academic Literature |
|  |  | Propose a navigation aid to ensure ethical and legal issues when using digital health technologies for COVID-19. | [16] | Academic Literature |
|  |  | Propose an evaluation framework to assess the feasibility and effectiveness aspects of DCTAs. | [17] | Academic Literature |
|  |  | Propose a framework for ethically justifiable design considering all stages of the system development life cycle. | [24] | Academic literature |
|  |  | Propose a socio-technical framework for DCTAs. | [13] | Academic literature |
|  |  | Propose a framework for assessing DCTAs design, their deployment and government’s principles. | [25] | Academic literature |
|  | Guidelines for the development and design of DCTAs | Describe ten core principles that should be followed for the DCTAs development lifecycle. | [7] | Regulations |
|  |  | Discuss Interoperable Digital Proximity Tracing (IDPT) protocol. | [32] | Report |
|  |  | Suggest an assessment of best practice principles achieved in HSE’s COVID Tracker app. | [35] | Report |
|  |  | Discuss principles for legislators on the implementation of new digital technologies in Ireland. | [36] | Report |
|  |  | Suggest survey results reported from the EU Member States and an overview of the progress made with the use of mobile contact tracing applications. | [37] | Report |
|  | Design requirements for the development and design of DCTAs | Suggest an EU Toolbox with essential requirements for developing effective DCTA solutions. | [6] | Published policy documents: EU legislation |
|  | Guidelines for the development and design of DCTAs | Describe the characteristics, design and functions that an effective DCTA should include. | [26] | Grey Literature |
|  | Characteristics of DCTAs | Discuss the centralised and decentralised approaches used for the contact tracing data. | [40] | Grey Literature |
|  |  | Classify three types of applications for COVID-19, e.g. those using exposure-notification Google /Apple API, those not using exposure-notification Google/Apple API, and apps for self-reporting of symptoms observed by the user. | [41] | Grey Literature |
|  |  | Identify four types of DCTAs based on the approach used for collecting contact tracing data: Bluetooth technology (first generation), Google-Apple Bluetooth approach,  GPS- phone location data and collect personal health data with other valuable information of the users. | [42] | Grey Literature |
|  |  | Describe four categories of COVID-19 contact tracing applications based on their design, e.g. Location, Bluetooth, Google/Apple and DT-3T. | [43] | Grey Literature |
|  |  | Describe the characteristics of DCTAs, e.g. centralised and decentralised as both use Bluetooth or GPS technologies for the contact tracing function. | [9] | Academic Literature |
|  | COVID-19 Exposure Notification | Discuss Apple /Google user interfaces and sample code for COVID-19 Exposure Notification in Apps. | [33] | Grey Literature |
|  |  | Discuss Apple’s Exposure Notification system for COVID-19 applications. | [34] | Grey Literature |
|  |  | Provide an overview of COVID-19 Exposure Notification when Using Bluetooth Low Energy. | [38] | Grey Literature |
|  |  | Discuss some of the functions available in the HSE COVID Tracker application (Ireland). | [39] | Grey Literature |
| **2 Mobile applications** | Evaluation frameworks for mHealth applications | Propose a hierarchical usability model consisting of 7 criteria and 23 sub-criteria for usability evaluation of mHealth applications. | [27] | Academic Literature |
|  |  | Propose a set of usability attributes and a framework for developing mHealth applications. | [44] | Academic Literature |
|  |  | Propose mHealth app trustworthiness checklist with the characteristics of mHealth apps should include. | [28] | Academic Literature |
|  |  | Suggest a tool for usability evaluation of mHealth applications with particular attention to universal and inclusive design approaches used for creating usable mobile apps. | [29] | Academic Literature |
|  |  | Offer guidelines for the design of mHealth applications by integrating Universal Design (UD) principles. | [45] | Academic Literature |
|  |  | Propose a detailed set of accessibility guidelines to assess and design accessible mobile applications. | [46] | Academic Literature |
|  | Evaluation frameworks of mobile applications | Suggests a set of usability attributes for mobile application identified through a systematic review. | [47] | Academic Literature |
|  |  | Discuss different usability factors of mobile applications. | [48] | Academic Literature |
|  |  | Suggest a catalogue with a set of usability guidelines for developing mobile applications and websites. | [49] | Academic Literature |
|  |  | Offer a systematic literature review of usability studies of mobile applications: definitions and attributes. | [50] | Academic Literature |
|  | Usability of interactive systems, taxonomy | Investigate usability concepts and suggest a detailed, hierarchically organised taxonomy for the development of usable technologies. | [51] | Academic Literature |
|  | Framework for an interactive interface for minors | Develop a framework for children to design touchscreen interface interaction: Touchscreen Interaction Design Recommendations (TIDRC). | [52] | Academic Literature |
|  | Guidelines for designing interactive systems for minors | Discuss guidelines for developing and evaluating applications for children at a different age. | [53] | Academic Literature |
|  | GDPR regulations for minors as users | Offer GDPR regulations for minors as users of information technology/services: parental/guardian consent (GDPR, Art 8). | [54] | Published policy documents: EU legislation |
|  | Guidelines for mobile application design | Present Directive (EU) 2016/2102 of the European Parliament and the Council on accessibility to ensure that the mobile applications developed for public bodies are accessible to all individuals with various physical and mental impairments. | [55] | Published policy documents: EU legislation |
|  | Requirements for accessible mobile application | Suggests accessibility requirements for ICT products and services. | [30] | Published policy documents: EU legislation |
|  | Accessibility guidelines for mobile application design | Provide information on the Web Content Accessibility Guidelines (WCAG) for designing websites and mobile applications. | [56] | Professional standards |
|  | Accessibility guidelines for mobile application design | Discuss guidelines for improving colour accessibility, particularly on designing the elements used in the UI for colour blind users. | [57] | Grey Literature |
|  | Guidelines for mHealth application | Offer guidelines for developing and evaluating mHealth applications, distributed into five categories: privacy, security, operability, content, and usability. | [31] | Grey Literature |

Table 2. Initial list of attributes extracted from identified 44 sources.

| **Sources** | **Attributes** |
| --- | --- |
| **Literature specific to CTAs**  [6,7,9,13-17,24-26,  32-43,58,59] | Purpose, Usability, Contact Tracing Definition, Updates, Data Controllers, Data Sharing Transparency, Core Functionality, User Consent, Interoperability, Information Accuracy, Organizational Attributes/Reputation, Transparency, Privacy, User Control/Self-Determination, Approved by National Authority, Legal Compliance, Public Ownership, Reporting (Positive COVID-19 Test), Voluntary, Scientific Validity, Non-discrimination, Repurposing, Systematic Accountability, Public Benefit, Expiration (Limited Purpose), Scalability, Data Collection, Backend Server, Preserved Users’ Anonymity, Data Protection, Data Management, Data Minimisation, Data Time Limitation, Securely Data Processing, Easy to Deactivate/Remove, Open-Source Code, Reliability, Explainability, Inclusiveness, Digital Inequality, Centralized, Decentralized, Alert Notification of Contacts, Using Google/Apple API, Not Using Google/Apple API, Bluetooth Technology, GPS- phone Location Data, DP-3T, WiFi-based Location Tracking |
| **Literature specific to Mobile and mHealth Applications, Universal Design, Accessibility, GDPR Regulations**  [27-31,44-57] | Effectiveness, Learnability, Memorability, Cognitive Load, Simplicity, Universality, Aesthetics, Security, Usefulness, Resources, Troubleshooting, Ongoing App Evaluation, Name of the Application, Navigation, Affordances, Interaction, Equitable Use, Flexibility, Ease of Use, Errors, Low Physical Effort, Size and Space for Approach and Use, Perceivable, Operability, Understandability, Robustness, Design, System, Content, Attractiveness, Comprehensibility, Accessibility, Consistency, Training, Trust, Battery Consumption, Less Storage Consumption, Adaptability, Performance, Layout, Platform Dependency, Onboarding, Speed, ISO 9241-11 (Efficiency, Satisfaction and Effectiveness), Parental/Legal Guardian Consent, Knowability, Clarity of the Elements, Clarity of the Structure, Clarity in Functioning, Helpfulness, Suitability of Documentation Content, Interactivity of Assistance, Completeness, Precision, Cultural Universality, Configurability, Workflow, Efficiency in Human Effort, Efficiency in Task Execution Time, Efficiency in Tied Up, Efficiency in Economic Costs, Safety, Subjective Satisfaction, Interest |

Table 3. Resulted grouping based on similar themes in existing frameworks and extracted attributes.

| **Pillars** | **Similar Themes in Existing Frameworks** | **Grouped Attributes** |
| --- | --- | --- |
| **Usability** | [14-17,27,29–31,44–50,52–56,58,61–63] | Usability, Updates, Core Functionality, Inclusiveness, Learnability, Memorability, Cognitive Load, Simplicity, Universality, Aesthetics, Ongoing App Evaluation, Navigation, Affordances, Interaction,  Equitable Use, Flexibility, Ease of Use, Errors, Low Physical Effort, Size and Space for Approach and Use, Perceivable, Operability, Understandability, Robustness, Design, Content, Attractiveness, Comprehensibility, Accessibility, Consistency, Adaptability, Layout, Onboarding, ISO 9241-11 (Efficiency, Satisfaction and Effectiveness), Parental/Legal Guardian Consent, Knowability, Clarity of the Elements, Clarity of the Structure, Clarity in Functioning, Helpfulness, Suitability of Documentation Content, Interactivity of Assistance, Completeness, Cultural Universality, Configurability, Efficiency in Human Effort, Efficiency in Task Execution Time, Efficiency in Tied Up, Subjective Satisfaction |
| **Data Protection** | [28,65–76] | Data Controllers, User Consent  Legal Compliance, Non-discrimination, Expiration (Limited Purpose), Data Protection, Data Time Limitation, Securely Data Processing, Reliability, Trust, Security, Safety |
| **Effectiveness** | [58,77] | Information Accuracy, Public Benefit, Effectiveness |
| **Transparency** | [25,28,66] | Data Sharing Transparency, Purpose, Transparency, Privacy, Voluntary, Repurposing, Preserved Users’ Anonymity, Data Minimisation, Easy to Deactivate/Remove, Open-Source Code, Explainability |
| **Technical Performance** | [78,79] | Backend Server, System, Resources, Troubleshooting, Battery Consumption, Less Storage Consumption, Performance, Speed, Precision |
| **Citizen Autonomy** | [16,28,58] | User Control/Self-Determination, Data Management |
| **Characteristics** | [32–39,78,80–85] | Contact Tracing Definition, Interoperability, Organizational Attributes/Reputation, Approved by National Authority, Public Ownership, Reporting (Positive COVID-19 Test), Scalability, Data Collection, Digital Inequality, Centralized, Decentralized, Alert Notification of Contacts, Using Google/Apple API, Not Using Google/Apple API, Bluetooth Technology, GPS- phone Location Data, DP-3T, WiFi-based Location Tracking, Name of the Application, Platform Dependency |
