# Supplementary figures and images for: Digital Contact Tracing Apps for COVID-19: Development of a Citizen-Centered Evaluation Framework
Source: JMIR Mhealth Uhealth. 2022 Mar 11;10(3):e30691. doi: 10.2196/30691 (PMC8919989; doi:10.2196/30691)

## Multimedia Appendix 2

A snippet of the Cross-pillar/ambiguity analysis matrix.


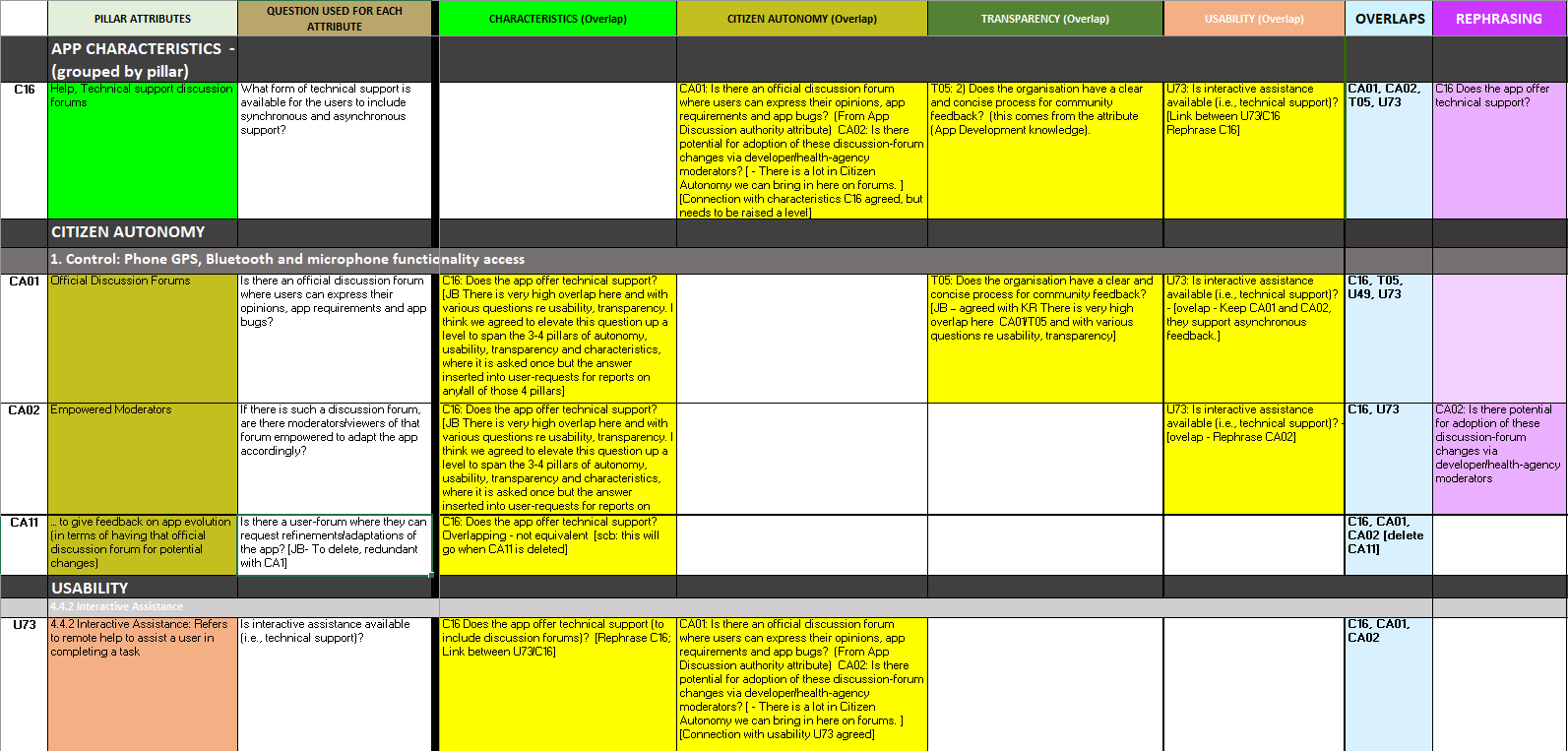

Supplement: Multimedia Appendix 2 [file mhealth_v10i3e30691_app2.docx]
