# Supplementary material for: Digital Contact Tracing Apps for COVID-19: Development of a Citizen-Centered Evaluation Framework
Source: JMIR Mhealth Uhealth. 2022 Mar 11;10(3):e30691. doi: 10.2196/30691 (PMC8919989; doi:10.2196/30691)
Supplement: Multimedia Appendix 3 [file mhealth_v10i3e30691_app3.pdf]

Multimedia Appendix 3

Visualization of all pillars and questions.

Citizen-Focused Compare-and-Contrast Evaluation Framework (C^3EF) for digital Contact Tracing Applications (CTAs) for COVID-19

The Citizen-Focused Compare-and-Contrast Evaluation Framework (C^3EF) is designed to help improve existing digital Contact Tracing Applications (CTAs) developed for COVID-19. The framework includes 7 pillars, e.g., Characteristics, Effectiveness, Technical Performance, Citizen Autonomy, Transparency, Data Protection and Usability. Each pillar has attributes, sub-attributes and at least one probing question.

CHARACTERISTICS	Captures non-judgemental criteria, e.g., app identifiers, organisation ids, functional overview.
EFFECTIVENESS	Measures the success of an app wrt its accuracy/detecting/sharing of contacts/its adoption by the population.
TECHNICAL PERFORMANCE	Focuses on system resource utilization and execution speed including processor working, both front-end and back-end.
CITIZEN AUTONOMY	Measures the degree to which a user can define their own comfort level in terms of rights/access they grant an app.
TRANSPARENCY	Involves questions unrelated to functionality and focuses on documentation of source code, privacy policy, etc.
DATA PROTECTION	Indicates the capacity to protect the rights of an individual during collection and processing of personal data.
USABILITY	Assesses the user interface and its ability to support satisfactory, accessible and consistent user interaction.

CHARACTERISTICS

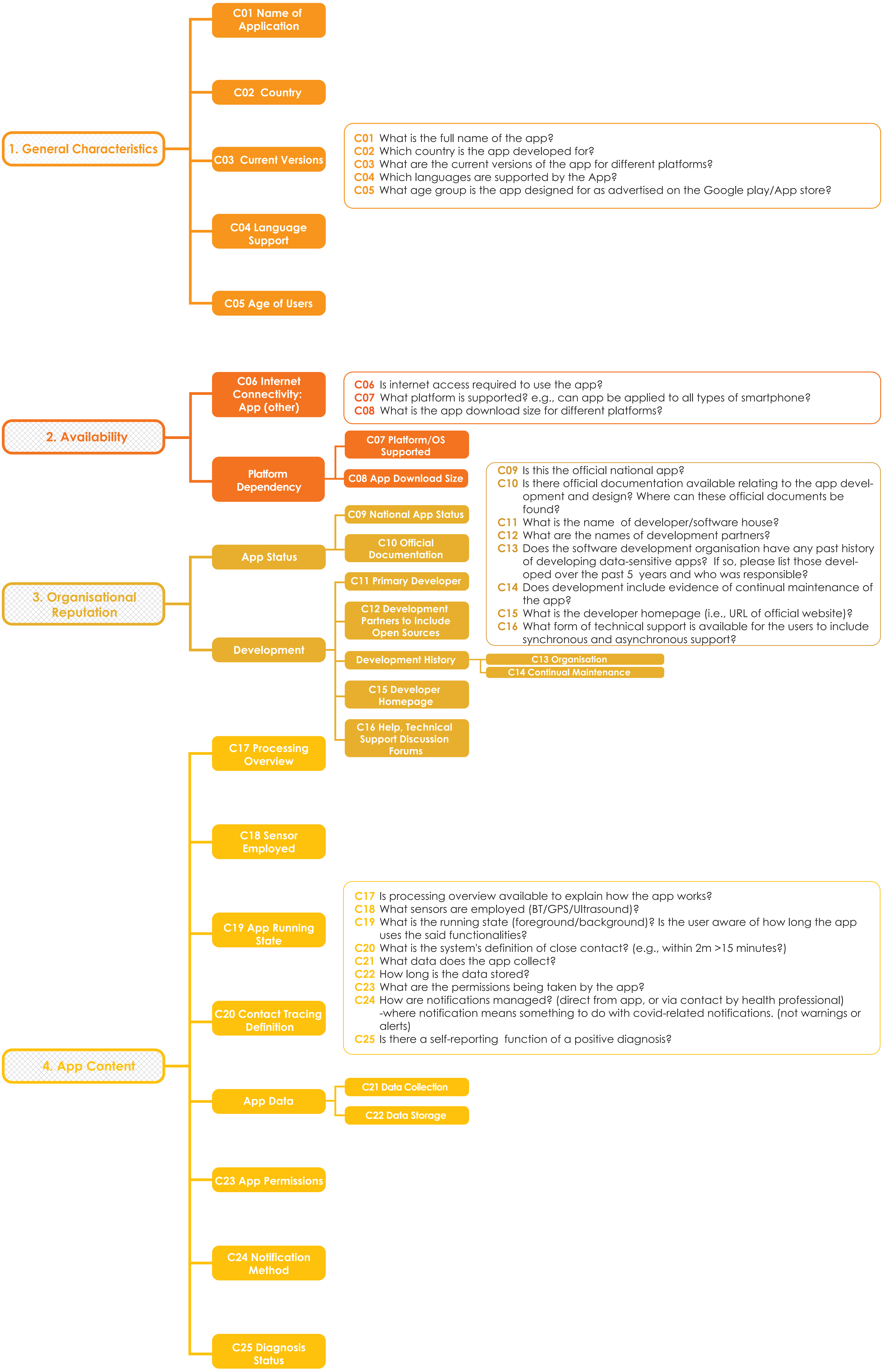

EFFECTIVENESS

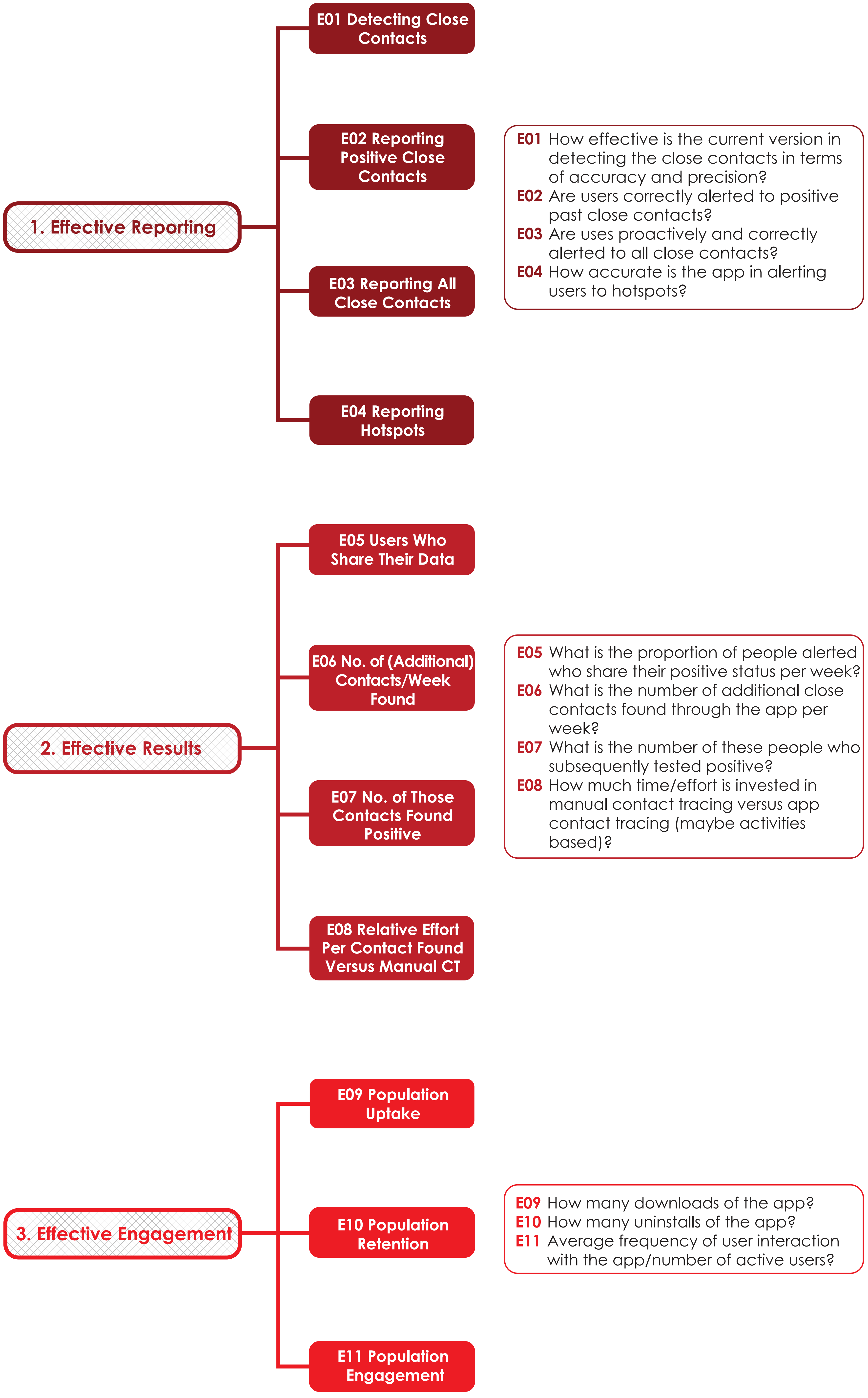

TECHNICAL PERFORMANCE

1. Speed

- **TP01 Response Time (Frontend)**

TP01 How fast (in ms) the frontend app responds to user interaction?

2. Efficiency

- **TP02 Response Time**

TP02 How fast (in ms) the algorithm(s) responds to user interaction?

3. Consumption

- **TP03 Battery**
- **TP04 Disk Space**

TP03 How much of a battery is consumed (%) by app per day/week/month?
TP04 How much of a disk space is used by an app?

4. Resources and Troubleshooting & Trust

- **TP05 CPU/Memory Usage**
- **TP06 Bandwidth Usage**
- **TP07 Throughput (Backend)**

TP05 How much of a CPU/memory is used by an app?
TP06 How much of a bandwidth is used by an app?
TP07 How many user requests a backend system can process per unit of time?

CITIZEN AUTONOMY

1. App Discussion Authority

- CA01 Official Discussion Forums
- CA02 Empowered Moderators

CA01 Is there an official discussion forum where users can express their opinions, app requirements and app bugs?
CA02 If there is such a discussion forum, are there moderators/viewers of that forum empowered to adapt the app accordingly?

2. Phone Functionality

- CA03 GPS Access
- CA04 Bluetooth
- CA05 ENS Access
- CA06 Notifications
- CA07 Microphone

CA03 Can the user back out of allowing the app access to the GPS phone functionality?
CA04 Can the user back out of allowing the app access to the Bluetooth phone functionality?
CA05 Can the user back out of allowing the app access to the ENS phone functionality?
CA06 Can the user back out of allowing the app access to the phone notification functionality?
CA07 Can the user back out of allowing the app to record the microphone?

3. Data Control

- CA08 Data Upload Authority
- CA09 Uploaded Data Location Visibility

CA08 Does the user explicitly control uploading of their medical-personal/GPS/Contact/Other data?
CA09 Is there user-available information to show them where their uploaded data resides?

TRANSPARENCY

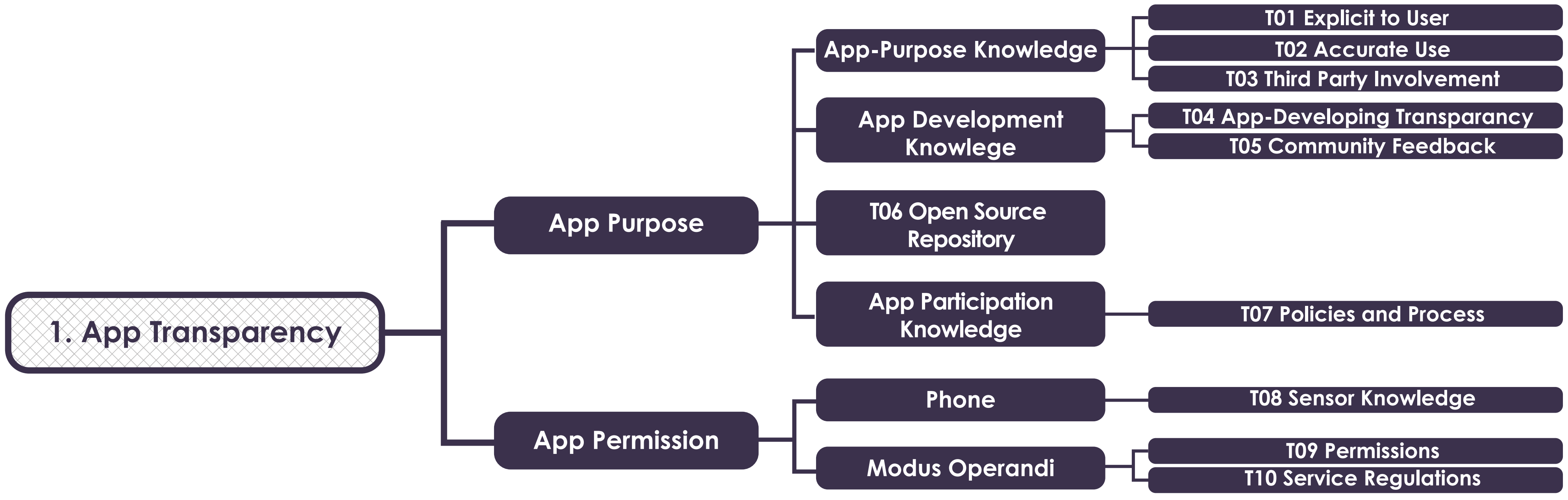

T01 Is the purpose of the app made accurately and accessibly explicit to the user?
T02 Is the app being used only for the purpose it has been stated?
T03 Is the app integrating any third-party software or using any third-party apps? If yes, then what are the apps?
T04 Are the app-developing companies/authorities made explicit to the users? Is reputational information accessible?
T05 Does the organisation have a clear and concise process for community feedback?
T06 Is the source code publicly available?
T07 Are the organisation's internal policies, staff, functions and development process visible?
T08 Does the app explicitly identify the phone functionalities it accesses (GPS, Bluetooth data) in a document format?
T09 Is the app being transparent about the necessity of permissions required for its functionality?
T10 Does the app regulate a time period over the services being used for its working?

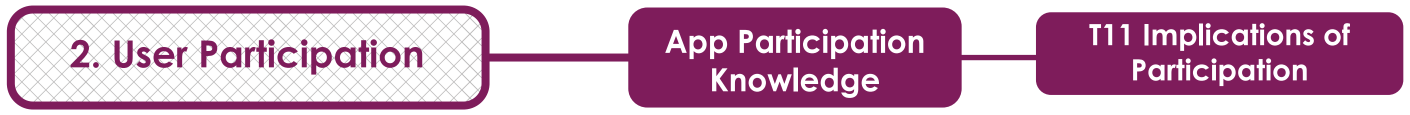

T11 Does the app indicate and explain to the end-user about the voluntary nature of participation?

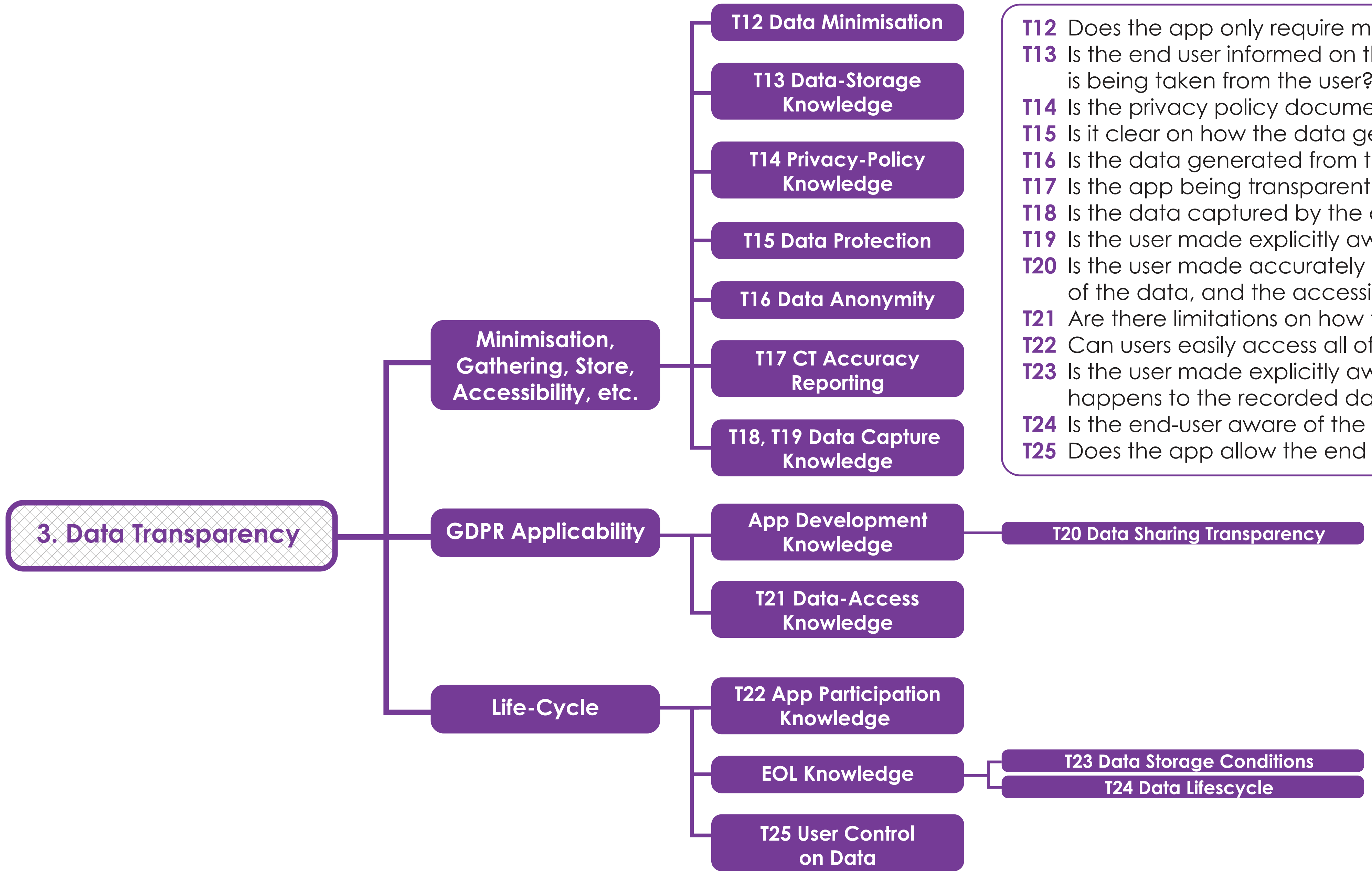

T12 Does the app only require minimal personal data of the end users (name, email address, location, etc.)?
T13 Is the end user informed on the data being collected by the app(i) in DPIA and (ii) when the information is being taken from the user?
T14 Is the privacy policy documented?
T15 Is it clear on how the data generated on the app is encrypted?
T16 Is the data generated from the app anonymised so that the individuals are non-identifiable?
T17 Is the app being transparent about the contact tracing accuracy that they are achieving?
T18 Is the data captured by the app made accurately and accessibly explicit to the user?
T19 Is the user made explicitly aware of where their data is stored (including jurisdiction) and for how long?
T20 Is the user made accurately and accessibly aware of the data accessible to other bodies, both in terms of the data, and the accessing bodies?
T21 Are there limitations on how the data gets used?
T22 Can users easily access all of their data?(A) via the app (B) by contacting the officials?
T23 Is the user made explicitly aware of the conditions deemed necessary for app end-of-life, and what happens to the recorded data at that point?
T24 Is the end-user aware of the life-cycle of data collected?
T25 Does the app allow the end user to delete their data?

DATA PROTECTION

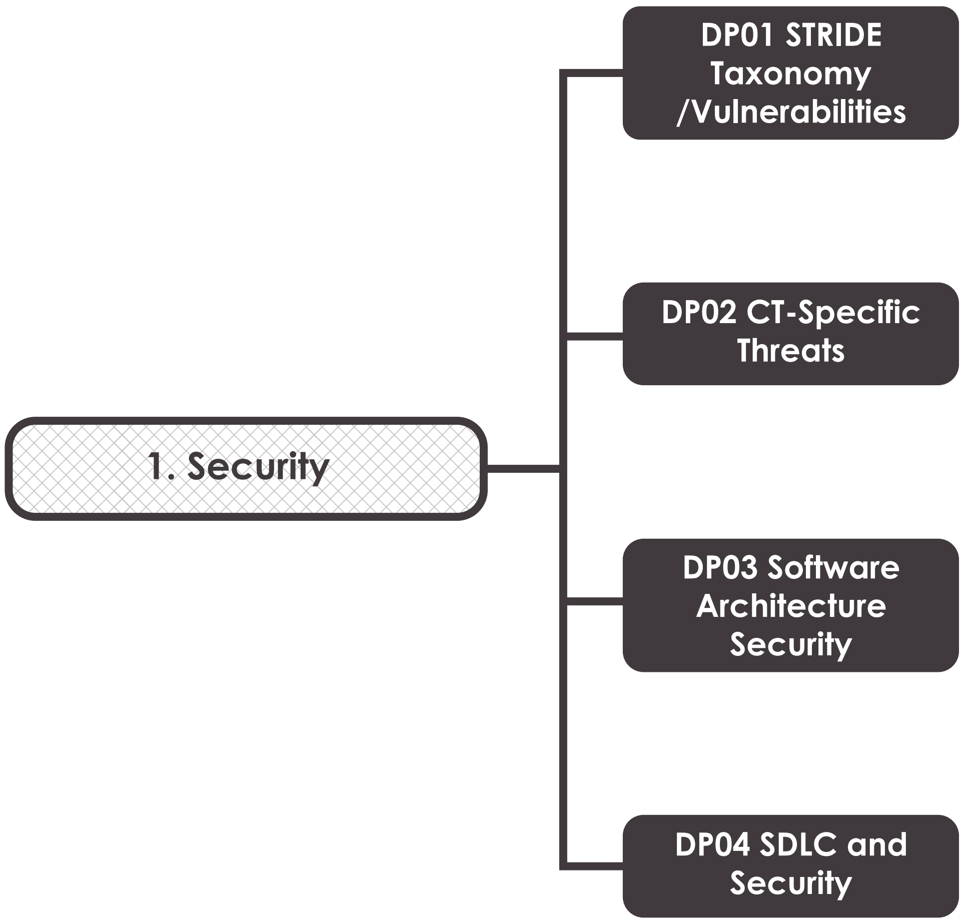

DP01 What obvious security vulnerabilities are present?
DP02 What contact-tracing specific threats are posed to this system?
DP03 How do these vulnerabilities relate to the software architecture?
DP04 How do the vulnerabilities of the software architecture affect its on going development?

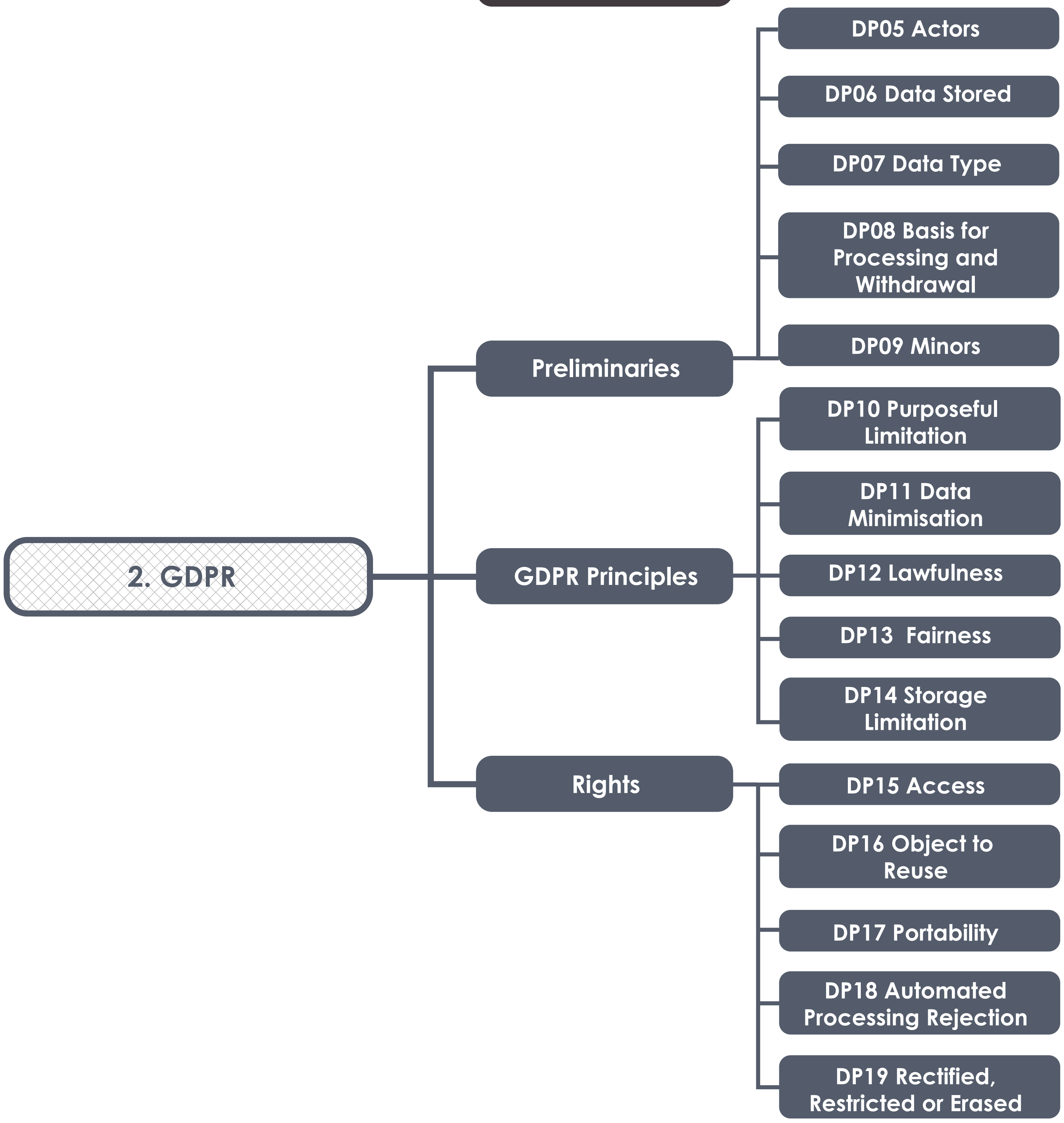

DP05 Who are the data controllers and processors?
DP06 What personal data is collected?
DP07 Is the data identifiable, and is it special category data?
DP08 What is the basis for processing the data? (legal, consent etc.)
DP09 Does the data involve or identify minors?
DP10 Is the data only being used for the purpose that it was collected?
DP11 Can less data be collected for the same purpose?
DP12 Is data collected legally? Does it identify criminal offense?
DP13 Are there any clear issues in which the data is not processed fairly?
DP14 Is data only stored for as long as necessary?
DP15 Is there a clear way for the user to access their data from the organisation in a specific time via subject access request?
DP16 Is the user able to reject to instances where their data is used for other purposes?
DP17 Is there a method that allows the user to retrieve their data in a portable format?
DP18 Can the user reject automated processing decisions?
DP19 Is there a clear way for the user to change, restrict or erase their data from the organisation? Does this process work?

USABILITY

1. Subjective Satisfaction

- U01 Rating
- U02 Motivations for High Score
- U03 Motivations for Low Score

U01 How would you rate your expe- rience with this app from 1 to 5?
U02 What are the main motiva- tions (reasons) for satisfied users?
U03 What are the main motiva- tions (reasons) for unsatisfied users?

2. Universality

- Accessibility
  - Functional Performance
    - Visual
      - U04, U05 Usage Without/or Limited Vision
      - U06, U07 Usage Without Perception of Colour
    - Auditory
      - U08 Usage Without/or Limited Hearing
    - Vocal
      - U09 Usage Without Vocal Capability
    - Motor
      - U10 Usage With Limited Manipulation or Strength
    - Cognitive
      - U11 Usage With Limited Cognition
    - U12, U13, U14, U15, U16, U17 Age/Parental Control
  - UI Elements
    - U18, U19, U20 Navigational /Interactive Elements
    - U21, U22, U23 Input Field
    - U24, U25, U26, U27 Text and Graphics
    - U28, U29 Content
  - Accessible Interactions
    - U30, U31 Onboarding
    - U32, U33, U34 Low Physical Effort
- Cultural Universality
  - U35 Name Qualities
  - U36 Language Activation
  - U37 Age/Terms and Conditions
  - U38 Cultural Conventions

U04 Does the app design accommodate the assistive technologies activated in the accessibility settings (e.g., screen readers)?
U05 Does the app provide an appropriate content layout for screen readers available on iOS and Android platforms (i.e., text for images, labels, icons and loading states)?
U06 Is a colour-blind friendly design available (i.e., descriptive labels of colours filters and swatches, underline links, the use of patterns and textures for graphs and charts, symbols to mark required form fields)?
U07 Are the used colours appropriate for colour blind users (e.g., colour combinations that should be avoided: green-red, green-blue, green-brown, green-black, green-grey, blue-grey, light green-yellow, blue-purple)?
U08 Are alternative options to interact with the app that does not require/or with limited hearing available?
U09 Can a user fully operate the app without the use of voice?
U10 Does the app accommodate a variety of input modalities, i.e., speech recognition, handwrit- ing recognition, gestures or use of an external keyboard? If yes, do these support users to validate inputs, i.e., confirmation screens or undo/redo options?
U11 Are there features which make the technology simple and easy to use by individuals with limited cognitive, language and learning abilities?

U12 Is a notice available for the suitability of the content for specific age groups?
U13 Is there a verification process available throughout the parental/legal guardian consent for children under the age of 13 (14 or 15) to minimise the collection, use, or disclosure of personal information/data from children's devices?
U14 Are mechanisms used to prevent deactivation of contact tracing function on the child's mobile device without the permission of the parents/- legal guardians?
U15 Are mechanisms used to prevent sending collected data on the child's mobile device without the permission of the parents/legal guardians?
U16 Does the app send an alert message to parents/legal guardians that the information (data) was sent to the health authorities/developers from their child's mobile device and they can request it to be deleted?
U17 Is a simpler design available for kids?
U18 Is labelling clear (i.e., tab bars, buttons)?
U19 Are navigational components prominent enough?
U20 What approaches are used for the navigation path, e.g., hamburger menu/hidden, tab bar, vertical navigation, buttons, plus button?
U21 Are the input fields easy to identify and fill in? Are other options available for the input field i.e., voice control, QR code?
U22 Are input fields 32-40px in height?
U23 Is the touch target size between 7 to 10mm (i.e., for Android at least 48 × 48dp and for iOS at least 44 x 44pt)?
U24 Is magnification available?
U25 Is the font size between 14 and 16 pixels? Does the text incorporate appropriate spacing allowance between the lines (e.g., 1.2 times the font height)?
U26 Is the contrast ratio of small text (i.e., below 14pt bold/18pt regular) at least 4.5:1 against its background?
U27 Is the contrast ratio of large-scale text (i.e., 14 pt bold/18 pt regular and up) and graphics at least 3:1?
U28 Is the content appropriate for the user population (i.e., avoid the use of jargon or acronyms related to clinical knowledge)?
U29 Does the content (e.g., functionality and readability) retain the same after the adjustments are made to accommodate accessibility?
U30 Does the app offer onboarding for new users?
U31 What approach was used for the design of onboarding, i.e., animations, video, text, images? If these are videos, animations and audios, is there transcripts, captions and/or audio descriptions available?
U32 Can a user complete a task without scrolling? If no, are the content elements critical to the app functionality positioned above the scroll line?
U33 Does the app support interaction with one-hand use (i.e., left or right)?
U34 Are select menus, radio buttons and checkboxes used?

U35 Is the name easy to recognise, to remem- ber and to find on Google Play and Apple Store? (e.g., appcovidhseie)
Does the name suggest any (false/mis- leading) sense of protection/security? (e.g., covid total protection app)
Does the name avoid any emphasis on the disease/infection/virus, which might cause social stigma? (e.g., personal infection tracker)
U36 What approach is used to activate the available language(s)?
U37 What age group is the app designed for as stated in the 'Terms and Conditions'? (Are they the same as advertised in app stores?)
U38 Are cultural conventions used in the design?

3. Design Effectiveness

- Completeness
  - U39 Core Functionality
  - U40 Optional Functionalities
  - U41 Completeness of Functionalities
- Configurability
  - Technical
    - U42 Access GPS
    - U43 Access Bluetooth
    - U44 Access ENS
    - U45 Access Phone Notification
    - U46 Access Microphone
    - U47 Connection Failure Feedback
  - Design
    - U48 Visual
    - U49 Acoustic
    - U50 Haptic
- User Interface
  - Aesthetic and Attractiveness
    - U51 Aesthetic
    - U52 Colour Scheme
  - U53 Responsiveness
  - Clarity (Visibility, Simplicity)
    - Clarity of Elements
      - U54 (ES), U55 (ES) Perceptual Clarity of Elements
      - U56 (ES) Conceptual Clarity of Elements
    - Clarity of Structure
      - U57 (ES) Perceptual Clarity of Structure
      - U58 (ES) Conceptual Clarity of Structure
  - Consistency
    - Consistency of Elements
      - U59 (ES) Perceptual Consistency of Elements
      - U60 (ES) Conceptual Consistency of Elements
    - Consistency of Structure
      - U61 (ES) Perceptual Consistency of Structure
      - U62 (ES) Conceptual Consistency of Structure
- Helpfulness
  - Suitability of Documentation
    - U63 Definitions
    - U64 Descriptions
    - U65 Examples
  - U66 Interactive Assistance

U39 What is the core functionality? (This question helps to identify the core functionality to which Task Specific (TS) questions should be applied.)
U40 What optional functionalities are offered? (This question helps to identify all of the optional functionalities to which Task Specific (TS) question could applied.)
U41 Are all the necessary functionalities for CTA available? Is there evidence that users would expect more/other functionalities?
U42 Does the app seek permission for access to the GPS phone functionality?
U43 Does the app seek permission for access to the Bluetooth phone functionality?
U44 Does the app seek permission for access to the ENS phone functionality?
U45 Does the app seek permission for access to the phone notification functionality?
U46 Does the app seek permission to record the microphone?
U47 Is any feedback available when the app fails to connect to any of these technologies?
U48 Does the app design accommodate the OS-level changes from the device settings (e.g., font, colour, contrast)?
U49 Does the app design support user configuration on acoustics?
U50 Does the app design support user configuration on haptics?
U51 Is the app aesthetically pleasing?
U52 Do the users like the colour scheme used in the user interface?
U53 Does the user interface and readability of content look different on different platforms?

U54 (ES) What are the elements used in the design of the interface? (This question helps to identify the elements for the Element Specific (ES) questions.)
U55 (ES) Are the interactive elements of the interface easy to identify?
U56 (ES) How easy is it to understand the meaning of the elements (what they do/are)? Can I tell what it is?
U57 (ES) Are the structures identifiable/visible? (Structures are organisations of elements. This question concerns the meaning, labelling, and structuring criteria used to group elements together (e.g., Do items in one menu belong to the same category?)
U58 (ES) Are the structuring/organising criteria understandable?(Structures are organisations of elements. This question concerns the meaning, labeling, and structuring criteria used to group elements together (e.g., Do items in one menu belong to the same category?)
U59 (ES) Are the same elements designed in different ways?
U60 (ES) Does the same element behave in different ways? Is the same element (e.g., button, menu) coherently designed throughout the app (e.g., Are all buttons round and embossed? Are all menus formatted the same)?
U61 (ES) Are ways to visually organise elements used incoherently?
U62 (ES) Are ways to organise elements used coherently?

U63 Are relevant definitions offered?
U64 Are relevant descriptions offered?
U65 Are examples offered?
U66 Is interactive assistance available (i.e., technical support)?

4. User Interaction

- Efficiency
  - U67 (TS) Human Effort
  - U68 (TS) Time
  - U69 Tied-up Resources
- Robustness
  - U70 Orientation
  - U71 (TS) Muliti-Tasking
  - U72 (TS) Flexibility/ Multimodality
  - U73 (TS) Adaptability
  - (TS) Errors
    - U74 (TS) Error Messages
    - U75 (TS) Error Recovery
    - U76 (TS) Action Reversibility
- Clarity of Interaction with Elements
  - U77 (TS) Interactional Clarity with Elements (Predictability)
  - U78 (TS) Clarity of Feedback
- Consistency of Interaction with Elements
  - U79 (TS) Inconsistency of Action on Elements
  - U80 (TS) Inconsistency of Feedback
  - U81 (TS) Constraints
- Alerts and Notifications Messages
  - U82 (TS) Allert Messages
  - U83 (TS) Notification Control
  - U84 (TS) Notification Messages
  - U85 (TS) Notifications Access and Visualisation

U67 (TS) How many steps does it take to complete the core task?
U68 (TS) How much time does it take to complete the main task?
U69 Does completing the task require the need of external resources (e.g., power, etc.)?
U70 Is portrait and/or landscape layout available?
U71 (TS)Can the same task 'a' be executed while carrying out other tasks with the same phone (e.g., using Bluetooth earphones)?
U72 (TS) Can the same task 'a' be executed in different ways/in more than one place (e.g., activate/deactivate the contact tracing function, activate/deactivate notifications, activate/deactivate Bluetooth, GPS, micrphone)?
(TS) Are shortcuts if applicable available?
U73 (TS) Can the same task 'a' be executed in different contexts (e.g., choose as pertinent: at dark, in a very noisy environ- ment, not looking?

U74 (TS) Are error messages available? Are these clear to the users? Are they actionable?
U75 (TS) Is error recovery (e.g., undo, redo) available?
U76 (TS) Are actions on elements reversible?

U77 (TS) I can tell what to do with it?
U78 (TS) Is the feedback adequate when interacting and using the app? Is feedback clear, prominent and timely? Is it actionable? Are there instances of confusion or lack of feedback?
U79 (TS) Are there any instances in which the same interactive element should be operated (interacted with) in different ways (e.g., are all buttons just for push- ing? If some are for holding, do these look different?)?
U80 (TS) Is the feedback consistent when interacting with elements of the same type? Are there instances of inconsistent feedback when interacting with elements?
U81 (TS) Are constraints in place to prevent errors? Are these effective?
(TS) Are there false or wrong constraints when interacting with the interface (e.g., inactive buttons, greyed menus, missing elements)?
U82 (TS) Are different types of alert or error messages available? Are they action- able?
U83 (TS) Does the app have built-in notifica- tion settings? Is control on the notifica- tions available in the app?
U84 (TS) Are notification messages clear and actionable?
U85 (TS) Can the user easily access contact tracing data for the last 14 days, can these be visualised?

5. Ongoing App Evaluation

- U86 Frequency of Upgrade

U86 How often is the app upgraded?

LEGEND

- Main Theme
- Attribute
- Elements Specific (ES) — An element can vary from a button to a menu, a slider or a table that are used in the design of the User Interface. The questions assess the design of individual elements of an interface (e.g., button, menu, etc.) and are **Element Specific** (**ES**).
- Task Specific (TS) — Some of the usability questions are **Task Specific** (**TS**) in that they are applicable to the execution of one specific task on the interface from beginning to end. TS questions are not applicable to the app as a whole.
